# 4-Arylthiosemicarbazide derivatives as a new class of tyrosinase inhibitors and anti-*Toxoplasma gondii* agents

**DOI:** 10.1080/14756366.2021.1931164

**Published:** 2021-06-02

**Authors:** Adrian Bekier, Lidia Węglińska, Agata Paneth, Piotr Paneth, Katarzyna Dzitko

**Affiliations:** a Department of Molecular Microbiology, Faculty of Biology and Environmental Protection, University of Lodz, Lodz, Poland; b Department of Organic Chemistry, Faculty of Pharmacy, Medical University of Lublin, Lublin, Poland; c Institute of Applied Radiation Chemistry, Lodz University of Technology, Lodz, Poland; d Institute Center for Research on Innovative Biobased Materials (ICRI-BioM) – International Research Agenda, Lodz University of Technology, Lodz, Poland

**Keywords:** Molecular docking, SAR analysis, thiosemicarbazides, *Toxoplasma gondii*, tyrosinase

## Abstract

We report herein anti-proliferation effects of 4-arylthiosemicarbazides, with a cyclopentane substitution at *N*1 position, on highly virulent RH strain of *Toxoplasma gondii*. Among them, the highest *in vitro* anti-*Toxoplasma* activity was found with the *meta*-iodo derivative. Further experiments demonstrated inhibitory effects of thiosemicarbazides on tyrosinase (Tyr) activity, and good correlation was found between percentage of Tyr inhibition and IC_50_*_Tg_*. To confirm the concept that thiosemicarbazides are able to disrupt tyrosine metabolism in *Toxoplasma* tachyzoites, the most potent Tyr inhibitors were tested for their efficacy of *T. gondii* growth inhibition. All of them significantly reduced the number of tachyzoites in the parasitophorous vacuoles (PVs) compared to untreated cells, as well as inhibited tachyzoites growth by impeding cell division. Collectively, these results indicate that compounds with the thiosemicarbazide scaffold are able to disrupt tyrosine metabolism in *Toxoplasma* tachyzoites by deregulation of their crucial enzyme tyrosine hydroxylase (TyrH).

## Introduction

*Toxoplasma gondii* (*T. gondii*) is an obligate intracellular protozoan parasite that infects birds and all mammals, including humans. Due to its unusually wide range of intermediate hosts, *T. gondii* is one of the most successful pathogens among eukaryotes and toxoplasmosis is one of the world’s most common latent infection^1^^,^^2^. Although largely asymptomatic, chronic toxoplasmosis can be especially harmful to newborns infected congenitally, immunocompromised persons, or patients receiving immunosuppressive therapy for organ transplantation^3–9^.

*T. gondii* exists in three infectious stages, such as oocysts (with sporozoites), tissue cysts (with bradyzoites), and rapidly proliferated tachyzoites, which form the parasitophorous vacuole (PV), a structure created by apicomplexan parasites in the host cells. Oocysts are only produced in the definitive hosts – members of the *Felidae* family (domestic cats and their relatives). Unsporulated oocysts are excreted in feline faeces of infected individuals. Each sporulated oocyst contains two sporocysts which hold four sporozoites. Even if a single sporulated oocyst is ingested or inhaled by the intermediate host, including all classes of warm-blooded vertebrates, sporozoites transform to tachyzoites, which localised in neural and muscle tissues including brain, eye, skeletal muscle, kidney, spleen, blood, liver, and heart tissue, and convert to bradyzoites enclosed in cysts that persist chronically^10^. In the case of infected pregnant women, tachyzoites can penetrate into the foetus *via* the bloodstream or placenta during the acute phase or reactivation of the infection. Ingestion of the tissue cysts in contaminated meat is also a source of infection, as bradyzoites transform back into tachyzoites upon entering a new host^11^.

The genome of *T. gondii* contains two genes, TgAaaH1 constitutively expressed in both tachyzoites and bradyzoites and TgAaaH2 whose expression is uniquely upregulated in bradyzoite cysts^12^. These genes encode tyrosine hydroxylase (TyrH)^12^ that promote catalysis of L-tyrosine conversion into L-3,4-dihydroxyphenylalanine (L-DOPA)^13^, the precursor of dopamine. Reports have shown that *T. gondii* TyrH is related to the dopamine levels in the host’s brain^14^. To address the role of *TgAaaH* genes in oocyst development, Sibley et al. and McFarland et al.,^15^^,^^16^ and others^17^ investigated the effect of *TgAaaH1/TgAaaH2* gene deletion and determined that the *TgAaaH2* gene is not connected with the dopamine-dependency of *Toxoplasma* infection. An alternatively proposed role of *TgAaaH* genes involves the promotion of dityrosine crosslinking in oocyst wall proteins^18^. Although the role for *TgAaaH1/TgAaaH2* genes in the tachyzoite or bradyzoite stages has not been reported thus far, close examination of L-tyrosine metabolism in *Toxoplasma* at these stages has revealed that they are both dependent on the addition of exogenous L-tyrosine for efficient growth. Additionally, the efficient production of PVs formation is limited in the absence of L-tyrosine^19^. Moreover, Parker et al.^20^ described a family of plasma membrane-localised amino acid transporters, named apicomplexan amino acid transporters (ApiATs), that are ubiquitous in apicomplexan parasites, including *T. gondii*. Functional characterisation of these transporters in *T. gondii* shows that several are important for intracellular growth at the tachyzoite stage of the parasite, which is related with acute infections. One such protein is the exchanger for aromatic and large neutral amino acids, with exhibit particular importance for L-tyrosine uptake pathway and amino acid homeostasis, which is critical for parasite virulence. The collective reports^12–20^ prove the concept that compounds that inhibit exogenous L-tyrosine uptake into *T. gondii* and/or participate in the disruption of the parasite L-tyrosine metabolism, have the potential to progress into vital medicines for the effective treatment of toxoplasmosis. Unfortunately, to date, there are no effective treatments against bradyzoites, and those medicines that target the tachyzoites (pyrimethamine [PYR] and sulfadiazine are the most effective) are associated with toxicity and hypersensitivity^21–27^. Other serious problems, such as drug resistance and incidence of relapses after discontinuation of therapy have been also reported^28^^,^^29^. Hence, novel, efficacious anti-*Toxoplasma* agents are indeed greatly needed.

**Scheme 1.**
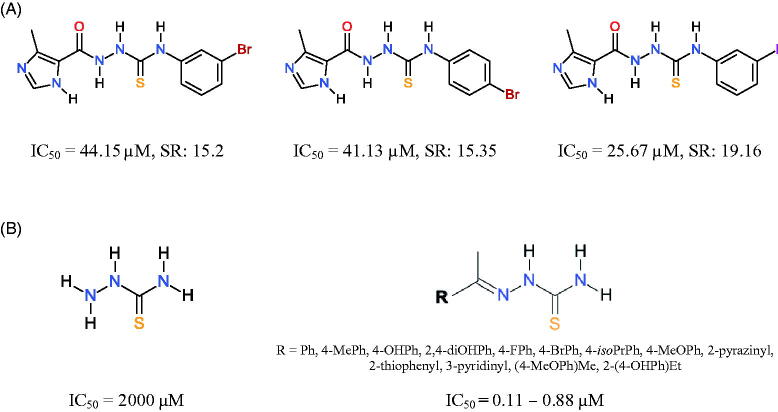
Structures of previously reported thiosemicarbazides and thiosemicarbazones with potent inhibitory activity against (A) *Toxoplasma gondii* proliferation^35^ and (B) tyrosinase^36^^,^^38^^,^^39^. SR – selectivity ratio, defined as the ratio of the 50% cytotoxic concentration (CC_50_) to the 50% antiparasitic concentration (IC_50_); sulfadiazine: IC_50_∼8500 µM, SR < 0.92; trimethoprim: IC_50_ 50.94 µM, SR > 1.20.

To address these issues, we explored the therapeutic potentials of low molecular weight heterocyclic compounds with thiosemicarbazide scaffold. In Liesen et al.^30^ on the example of small series of imidazole-thiosemicarbazides, reported for the first time anti-*T. gondii* potential for thiosemicarbazide-based compounds. The tested compounds showed better LD_50_ values for both infected cells and intracellular parasites than the standard drugs sulfadiazine and hydroxyurea. Inspired by those promising results, as well as those reported for closely related thiosemicarbazones^31^^,^^32^ efforts have been made by our research group to expand these initial findings with further details on anti-*T. gondii* activity of thiosemicarbazides-based compounds. Based on the systematic search,^33–35^ several promising candidates (Scheme 1(A)) were identified to inhibit RH-strain tachyzoites proliferation *in vitro* more effectively than standard drugs sulfadiazine or trimethoprim (TRI) and at the same time did not inhibit the growth of human cell lines, suggesting that the anti-*Toxoplasma* strategy can be non-toxic towards mammals. However, crucial information regarding the molecular mechanisms involved in their anti-*Toxoplasma* effect has yet to be reported. One of the key steps in determining the cause of therapeutic intervention is through the identification of the essential pathways that can be targeted by small molecules. Recent reports have shown the inhibitory activity of thiosemicarbazide and thiosemicarbazones against tyrosinase (Tyr) (Scheme 1(B)) which, similarly to TyrH, catalyses two initial sequential oxidations of L-tyrosine to L-DOPA^36–39^. Based on these results, we hoped that thiosemicarbazide-based compounds could show an inhibitory effect against *T. gondii* TyrH. Results of such studies have not been reported in the literature thus far. Using combined experimental and theoretical approaches, we hypothesised that the inhibitory effect of studied thiosemicarbazides on invasion and division of tachyzoites in host cells is related to their disruption of L-tyrosine metabolism in *Toxoplasma* tachyzoites and/or defects in exogenous L-tyrosine transport to parasitic cells.

These studies, described here, reflect our approach to support the hypothesis that the biological effect of the examined compounds results from disruption of *T. gondii* L-tyrosine metabolism by inhibiting TyrH. First, the problem of cytotoxicity of the studied compounds is addressed. Second, we measured the effect of the studied compounds on *in vitro* anti-*T. gondii* activity. Once the prepared compounds are determined as non-toxic and biologically active, we investigated if their effect on *T. gondii* is related to inhibitory activity against TyrH. However, this is a two-step process owing to the lack of access to enzyme preparations. Therefore, following literature precedents^40^, we used commercially available Tyr, which also catalyses the conversion of L-tyrosine to L-DOPA, as a proxy to the inhibition pattern of TyrH. Subsequently, a series of experiments screened for the inhibition of Tyr and its comparison with that of *T. gondii* proliferation *in vitro*. In order to evolve and strengthen the conclusion to our hypothesis, studies conducted on Tyr inhibition allowed for the interpretation of TyrH mechanism of action. Additionally, docking studies were performed on all compounds to both enzymes and showed the correspondence of the energetics of binding between them, as well as with *T. gondii* proliferation inhibition. In this case, we focussed on four compounds (**2a**, **2b**, **6a**, and **6b**), which exhibit the highest inhibitory activity against Tyr. To further understand the mechanism of inhibition we comprehensively investigated the kinetics of inhibition followed by docking studies. These studies highlighted binding modes and further supported the assumed correspondence between inhibition of Tyr and TyrH. Hence, we examined the efficacy of compounds **2a**, **2b**, **6a**, and **6b** in inhibiting *T. gondii* growth *in vitro* into human foreskin fibroblast cells using confocal microscopy.

## Results and discussion

**Scheme 2.**
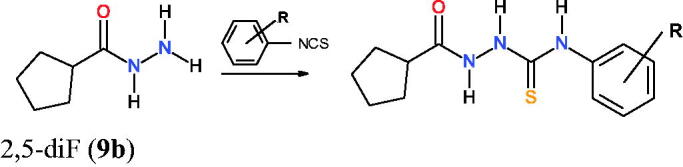
Synthetic route for set of cyclopentane-thiosemicarbazides **1b**–**9b**. R = *m*-NO_2_ (**1b**), *p*-NO_2_ (**2b**), *o*-Cl (**3b**), *o*-Br (**4b**), *o*-I (**5b**), *m*-I (**6b**), *o*-OMe (**7b**), *p*-Me (**8b**), and 2,5-diF (**9b**).

Several promising candidates with thiosemicarbazide scaffolds have been shown to inhibit RH tachyzoites proliferation *in vitro* more effectively and selectively than antifolate drugs (sulfadiazine, TRI, or PYR)^33–35^. Unfortunately, no structural basis or molecular mechanisms of underlying anti-*T. gondii* activity of thiosemicarbazide has been reported thus far in literature. Hence, a series of previously reported 4-arylthiosemicarbazides bearing N1 imidazole ring was examined^34^^,^^35^ (**1a**–**9a**, Table 1), which have proven non-toxic, as well as exhibited enhanced potency and selectivity compared to TRI, sulfadiazine, and combination. Additionally, to start the SAR exploration, we have also chosen to replace the N1 imidazole with more hydrophobic N1 cyclopentane to obtain a set of cyclopentane-thiosemicarbazides **1b**–**9b** (Table 1). These compounds were prepared using reported methods^33–35^ *via* a one-step reaction between cyclopentanecarbohydrazide and appropriate aryl isothiocyanate (Scheme 2). Detailed procedures for the synthesis and characterisation of compounds **1b**–**9b** are presented in the Experimental Section.

**Table 1.** The cytotoxic concentration (CC_30_), inhibitory concentration (IC_50_*_Tg_*), and selectivity ratio (SR) of compounds **1a**–**9a** and **1b**–**9b**.

Compound	CC_30_^a^	IC_50_*_Tg_*^b^	SR^c^	Compound	CC_30_^a^	IC_50_*_Tg_*^b^	SR^c^
**1a**	440.95	46.14	9.56	**1b**	616.52	179.95	3.43
**2a**	682.98	67.49	10.12	**2b**	378.39	144.05	2.63
**3a**	462.12	114.96	4.02	**3b**	554.73	367.70	1.51
**4a**	579.61	78.06	7.43	**4b**	806.60	218.09	3.70
**5a**	253.17	50.62	5.00	**5b**	333.25	127.99	2.60
**6a**	213.27	25.67	8.31	**6b**	117.68	54.49	2.16
**7a**	452.06	108.43	4.17	**7b**	886.29	326.57	2.71
**8a**	361.73	198.99	1.82	**8b**	463.37	181.41	2.55
**9a**	509.11	172.50	2.95	**9b**	860.66	321.11	2.68
Trimethoprim (**TRI**)	61.25	50.94	1.20				
Pyrimethamine (**PYR**)	181.97	11.48^d^	15.85				
Kojic acid (**KA**)	1558.28	501.19	3.11				

^a^CC_30_ [µM] represents the non-cytotoxic concentration of the compound required for 30% of cells proliferation inhibition *in vitro*. CC_30_ values were determined based on the plotted curves.

^b^IC_50_*_Tg_* [µM] represents the concentration of the compound required for 50% inhibition of *T. gondii* proliferation *in vitro*. IC*_50Tg_* values were determined based on the plotted curves using GraphPad Prism.

^c^SR represents selectivity ratio values were calculated as the ratio of 30% cytotoxic concentration (CC_30_) to 50% antiparasitic concentration (IC_50_*_Tg_*).

^d^IC_50_*_Tg_* of pyrimethamine in this study (11.48 µM) corresponds to the values (range 8.7–58.26 µM^41–43^) obtained in other studies using RH strain of *T. gondii*.

First, we evaluated the cytotoxic effects of **1b**–**9b**, which is necessary prior to measuring anti-*Toxoplasma* and Tyr inhibition in order to exclude cytotoxic compounds. The cytotoxic effects of **1a**–**9a** have been reported previously^34^^,^^35^. Cytotoxicity was estimated using mouse fibroblast (L929) cells line *via* MTT assay, according to the international standards ISO 10993–5:2009(E). *T. gondii*, an intracellular parasite, only multiplies inside the PV in the infected host cells, hence, host cells should not be destroyed by the cytotoxic concentration of the studied compounds. Therefore, the obtained results were expressed as CC_30_, which was defined as the highest concentration of the compound that causes 30% damage of cells and was considered according to ISO as non-toxic. CC_30_ values of cyclopentane-thiosemicarbazides **1b**–**9b** are presented in Table 1. CC_30_ values of imidazole-thiosemicarbazides **1a**–**9a** are also included for comparison.

As shown in Table 1, cyclopentane-thiosemicarbazides **1b**–**9b** did not show significant cytotoxic effect on L929 cells after 24 h of incubation and are considered good candidates for biological assays. The analysis of MTT results (e.g. **1a** *vs.* **1b**) also reveals that, except for compounds **2b** and **6b**, cyclopentane-thiosemicarbazides **1b**–**9b** show less cytotoxic effect than their N1 imidazole analogues **1a**–**9a**. It is worth noting that CC_30_ values for compounds **1b**–**9b** are at least 1.92–14.47 higher than the cytotoxic concentration of TRI and, except for compound **6b**, that at least 1.83 to 4.87 higher than the cytotoxic concentration of PYR.

At this point, we questioned if replacing N1 imidazole in the basic structure of 4-arylthiosemicarbazide by more hydrophobic N1 cyclopentane influences the anti-*Toxoplasma* effect. This strategy is based on observation that incorporation of more lipophilic substituent into thiosemicarbazide core structure improves anti-*T. gondii* activity^32^. To address this, we submitted **1b**–**9b** to *T. gondii in vitro* proliferation assay. PYR commonly used for the treatment of human toxoplasmosis, and TRI, and used to treat animals in Europe^44^, were employed as positive controls, whereas 0.1% concentration of dimethyl sulfoxide (compounds and drugs solvent) was used as the negative control (data not shown). In addition, anti-*T. gondii* activity of the reference Tyr/TyrH inhibitor, kojic acid (KA), was established. IC_50_*_Tg_* and selectivity ratio (SR) values of cyclopentane-thiosemicarbazides **1b**–**9b** and KA are presented in Table 1. IC_50_*_Tg_* values of imidazole-thiosemicarbazides **1a**–**9a** reported previously^34^^,^^35^ are also listed for comparison.

Although cyclopentane-thiosemicarbazides **1b**–**9b** show lower potency and selectivity in inhibiting *T. gondii* proliferation *in vitro* compared with more hydrophilic precursors **1a**–**9a**, they possess enhanced selectivity compared to TRI. Furthermore, consistent with data obtained for compounds **1a**–**9a**, *meta*-iodo compound **6b** (54.49 µM) is the most effective; it showed efficacy close to that of TRI (50.94 µM) and, at the same time, better selectivity (2.16 *vs.* 1.2). The presence of other substituents, both electron withdrawing and electron donating, also reduced potency most notably *ortho*-chloro **3b**, *ortho*-methoxyl **7b**, and 2,5-fluoro **9b**. Within the series of compounds **1b**–**9b** the activity follows the trend *m*-I (**6b**) > *o*-I (**5 b**) > *p*-NO_2_ (**2b**) > *m*-NO_2_ (**1b**) > *p*-Me (**8b**) > *o*-Br (**4b**) > 2,5-diF (**9b**) > *o*-OMe (**7b**) > *o*-Cl (**3b**), whereas activity of compounds **1a**–**9a** follows a slightly different trend *m*-I (**6a**) > *m*-NO_2_ (**1a**) > *o*-I (**5a**) > *p*-NO_2_ (**2a**) > *o*-Br (**4a**) > *o*-OMe (**7a**) > *o*-Cl (**3a**) > 2,5-diF (**9a**) > *p*-Me (**8a**).

Once the cytotoxic profile and *in vitro* biological activity of compounds **1a**–**9a** and **1b**–**9b** on *T. gondii* were determined, we subjected the studied compounds to anti-Tyr activity assay to establish the role of thiosemicarbazides, towards the activity of *Toxoplasma* TyrH. Since there is currently no commercial *Toxoplasma* TyrH, following literature precedents^40^ we used in our experiments mushroom Tyr that, similarly to TyrH, catalyses two initial sequential oxidations of L-tyrosine to L-DOPA.

**Figure 1.**
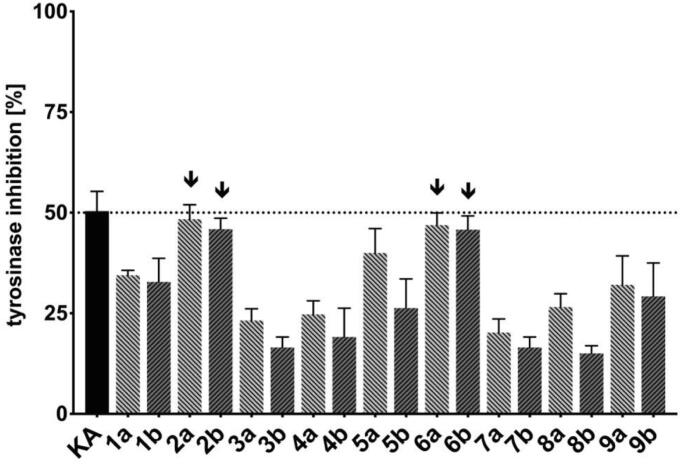
Tyrosinase inhibition results (% inhibition at 25 µM) for compounds **1a**–**9a**, and **1b**–**9b**, and **KA**. Data shown are the combined averages of three independent experiments in triplicate using the same conditions. Error bars indicate standard error of the mean. Values with an activity close to that of KA are labelled with arrows.

The reference inhibitor, KA, was used for the enzyme assay at the concentration of 25 µM at which 50% inhibition of Tyr was obtained in the preliminary tests (data not shown). A few general conclusions arise from this inhibitory study, as graphically summarised in Figure 1. Importantly, all studied compounds display efficacy below their cytotoxic concentration. Imidazole-thiosemicarbazides **1a**–**9a** universally produce higher inhibitory effect than their N1 cyclopentane substituted **1b**–**9b** analogues (e.g. **1a** *vs.* **1b**; Figure 1), which is in agreement with *T. gondii* proliferation measurements data. The observed differences may arise from the electronic effect of N1 imidazole on the whole thiosemicarbazide skeleton. Additionally, it may also be possible that the imidazole present in compounds **1a–9a** promote structural benefits either by providing increased hydrophilicity to adopt favourable conformations in the active site or favourable interactions between the imidazole and enzyme. Furthermore, among the thiosemicarbazides tested, *meta*-iodo **6a** (48.9 ± 3.0%) and **6 b** (43.9 ± 3.4%), and *para*-nitro **2a** (46.4 ± 3.6%) and **2b** (45.6 ± 2.8%) display the highest inhibitory effects, similar to that of KA (50.4 ± 4.9%), thus suggesting that the presence of the electron-withdrawing substitution at the *meta* or *para* position of N4 phenyl ring and (or) steric effect of the substituents can induce improved anti-Tyr activity. In addition, among *ortho*-halogen compounds, the steric and/or electronic change from chloro (**3a, 3b**) to bromo (**4a, 4b**) to iodo (**5a, 5b**) increase inhibitory potency. Although the precise role of the electronegativity of *ortho*-halogens in anti-Tyr activity remains unclear, changes in p*K*a or enhancement in binding created through decreased positive charge of N4 phenyl ring may be a possibility. Finally, the trend of inhibitory potency; *meta*-iodo **6a** (48.9 ± 3.0%) > *meta*-iodo **6b** (47.9 ± 3.4%) > *para*-nitro **2a** (46.4 ± 3.6%) > *para*-nitro **2b** (45.6 ± 2.8%), can be related to the substitution pattern and (or) steric effect of the substituents.

**Figure 2.**
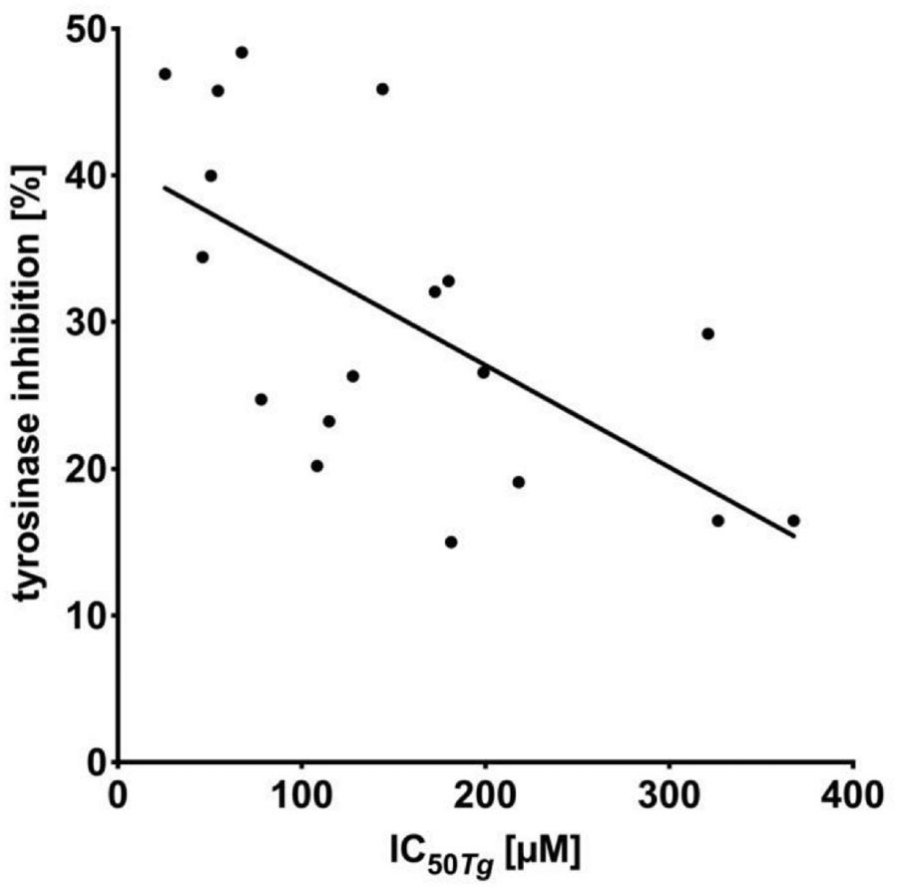
The correlation between the concentration of the compound required for 50% inhibition of *T. gondii* proliferation *in vitro* (IC_50_*_Tg_*) and tyrosinase inhibition % for compounds **1a**–**9a** and **1b**–**9b** (*r* = 0.84; *p* = .0053). The statistical relationships were analysed using Pearson correlation.

According to the obtained results from the *T. gondii in vitro* proliferation assay, good correlation exists (Figure 2, Pearson correlation coefficient *r* = 0.84; *p* = .0053) between percentage of Tyr inhibition and IC_50_*_Tg_* of both sets of compounds. This further supports the assumption that anti-*Toxoplasma* effect of thiosemicarbazides might be related to inhibition of Tyr, and thus, TyrH activity.

**Figure 3.**
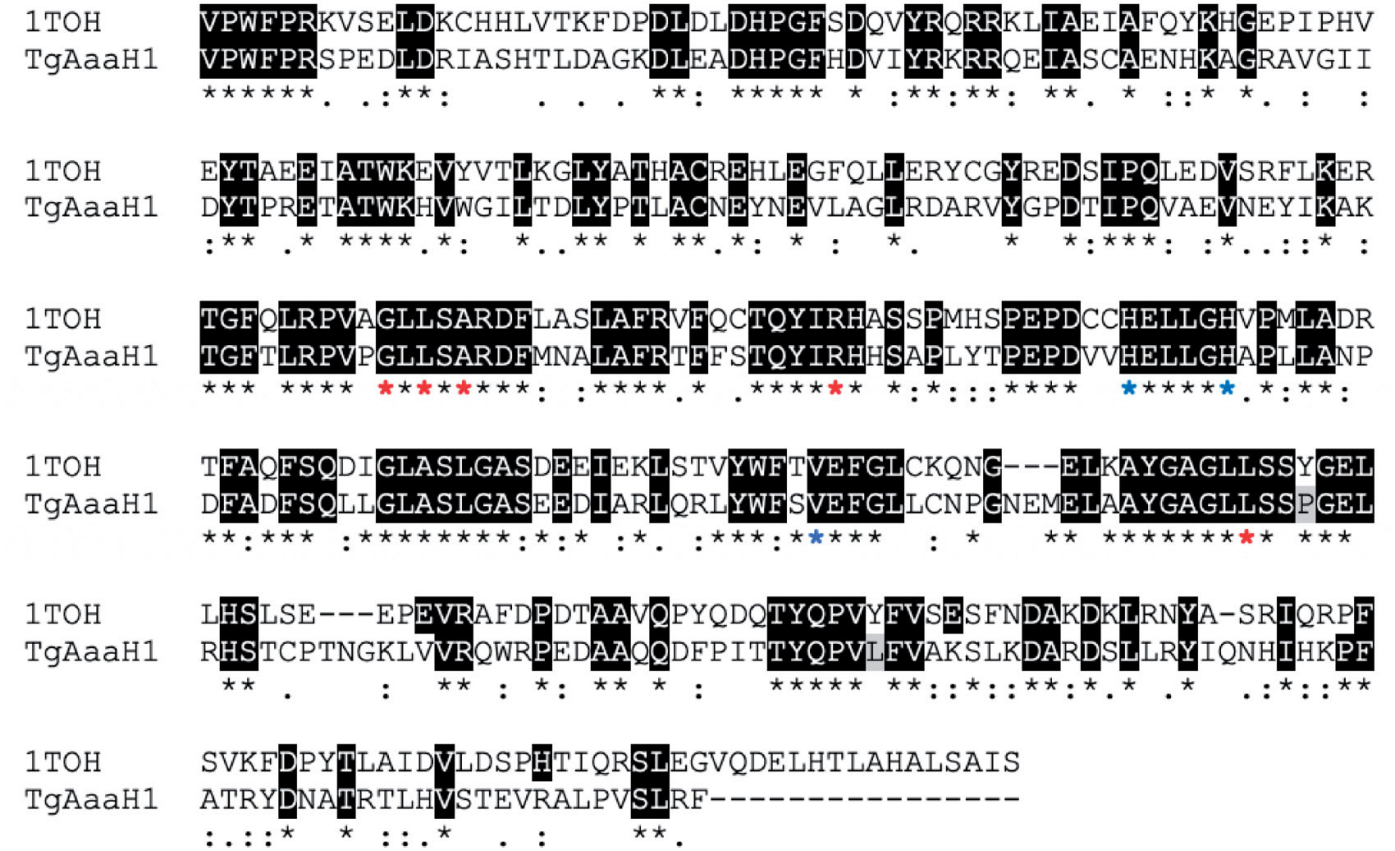
Alignment of aromatic amino acid hydroxylases generated using MUSCLE alignment software^44^. Identical residues between 1TOH and TgAaaH1 are highlighted in black. The positions that have a single and fully conserved residue are marked with an asterisk (*). The conservation between residues of strongly similar properties are marked with a colon (:), while conservation between residues of weakly similar properties are marked with a period (.). The iron-binding residues are highlighted with blue asterisk (*). The residues present in the active site that interact with the aromatic amino acid substrate and biopterin cofactor are highlighted with red asterisk (*)^12^.

**Figure 4.**
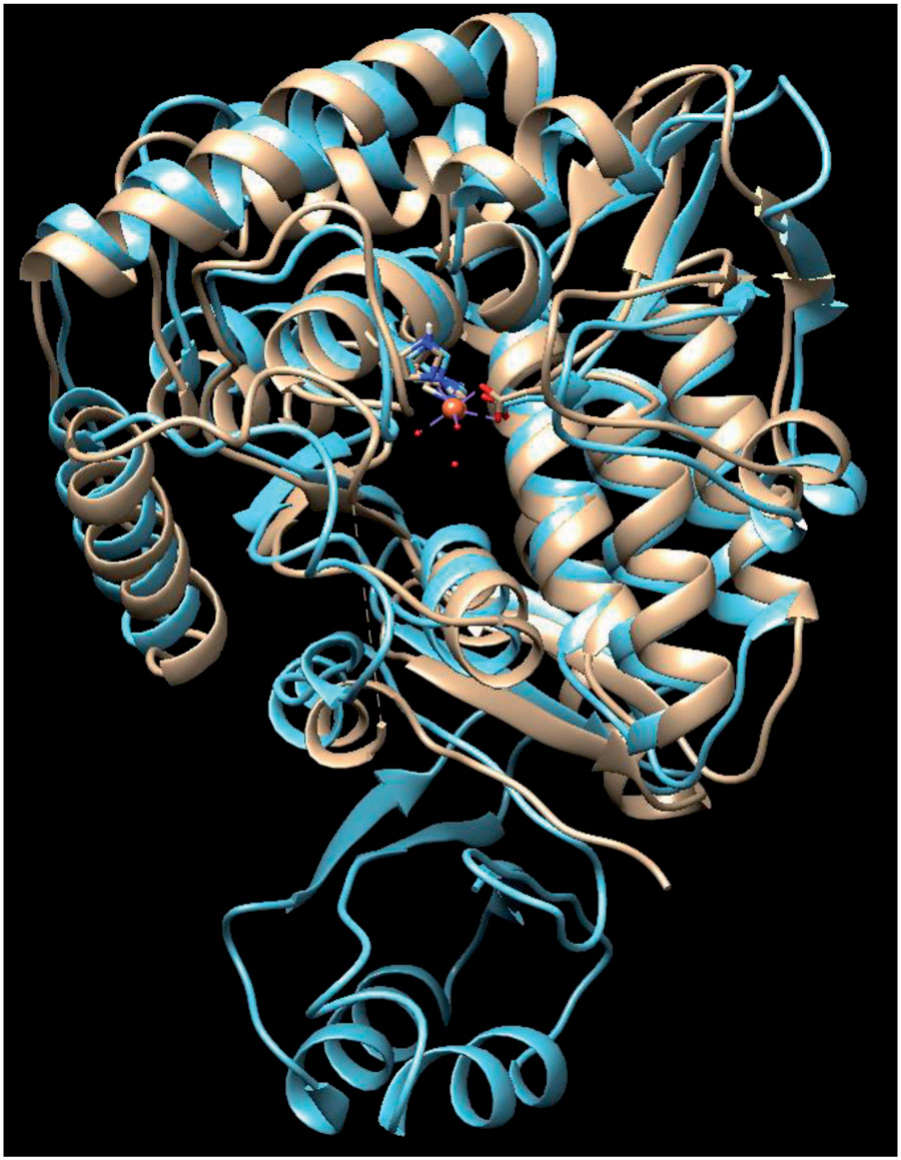
Comparison of 3D catalytic domains of tyrosine hydroxylases from rat (1TOH in gold) and *T. gondii* (AaaH1 in blue).

In order to test if Tyr inhibitors also act as *T. gondii* TyrH inhibitors, complementary docking studies were carried out. Currently, there is no reported 3 D structure of *T. gondii* TyrH, hence, the 3D structure of rat TyrH (PDB ID: 1TOH)^45^ is employed since its catalytic domains from rat and *T. gondii* are highly comparable^12^. As shown in Figure 3, the sequence of TgAaaH1 (UniProtKB code B2L7T1) in the catalytic domain is highly homological with rat enzyme and their 3D structure similarity is illustrated by Figure 4. The catalytic domain contains conserved VPWFPR motif that begins at V240 of the *T. gondii* sequence and residue V164 of the rat TyrH. This catalytic domain also contains conserved iron-binding residues at positions His407, His412, and Glu452, which correspond to His331, His336, and Glu376 in rat TyrH, respectively^46^. In addition, validity of structural modelling of *T. gondii* catalytic domains using the crystal structure of rat TyrH proxy is corroborated by the sequence alignment and placement of key residues (Figure 3 shows the alignment generated using MUSCLE software^47^). Therefore, our computational studies of the crystallographic structure of rat TyrH (PDB ID: 1TOH) were used.

**Figure 5.**
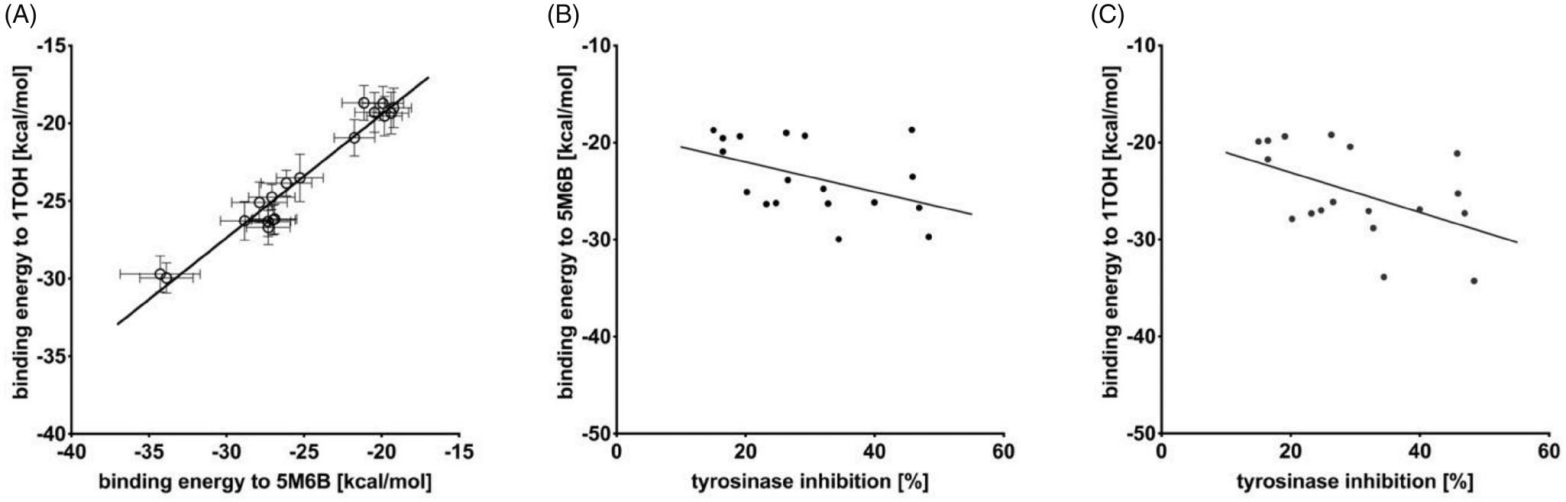
(A) The correlation between the binding energies calculated for 5M6B and 1TOH with the same set of ligands (**1a**–**9a** and **1b**–**9b**) (*r* = 0.98; *p* < .0001). Correlation between the % of tyrosinase inhibition and binding energies to (B) 5M6B (*r* = 0.76; *p* = .0438) and 1TOH (C) (*r* = 0.78; *p* = .0377). Statistical relationships were analysed using Pearson correlation.

First, in order to successfully predict the binding affinities to the active site of *T. gondii* TyrH, compounds **1a**–**9a** and **1b**–**9b** were docked to the active site of TyrH (PDB ID: 1TOH) and its proxy (for which experiments were performed) mushroom Tyr (PDB ID: 5M6B)^48^. As shown in Figure 5(A), predicable good correlation is observed (Pearson correlation coefficient *r* = 0.98; *p* < .0001) between the binding energies to 5M6B plotted and those predicted for 1TOH. Additionally, good correlation is observed between the binding energies of 5M6B or 1TOH plotted against the percentage of Tyr inhibition (Figure 5(B,C), respectively). The strong correlation between binding energies of compounds **1a**–**9a** and **1b**–**9b** to Tyr and TyrH indicate a similar mode of binding in both enzymes which further validates the implementation of the experimental results obtained for the Tyr in the interpretation of TyrH mechanism.

**Figure 6.**
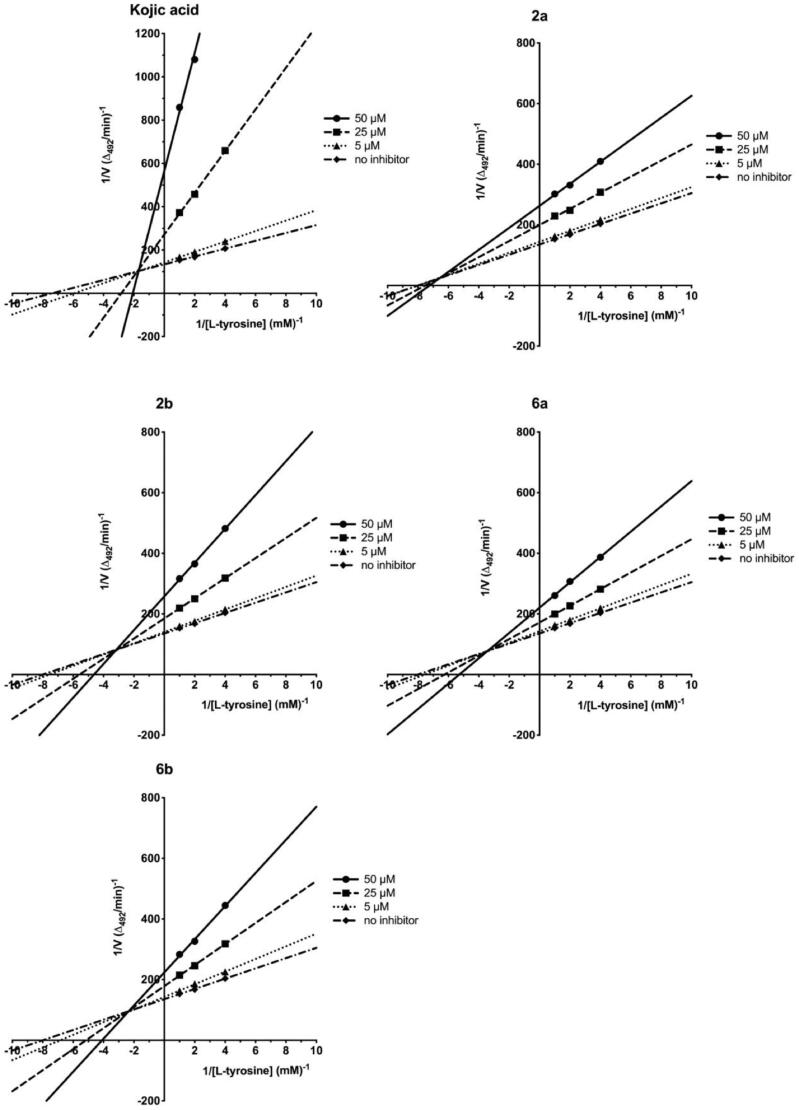
Lineweaver–Burk linearised plots with varying concentrations of compounds **2a**, **2b**, **6a**, **6b**, and **KA**. Concentration of inhibitor varied from 0 to 50 μM. Three concentrations of L-tyrosine as a substrate at 0.25, 0.5, and 1 mM were used. V – reaction rate. Each point represents the combined averages of three independent experiments in triplicate under the same conditions.

**Figure 7.**
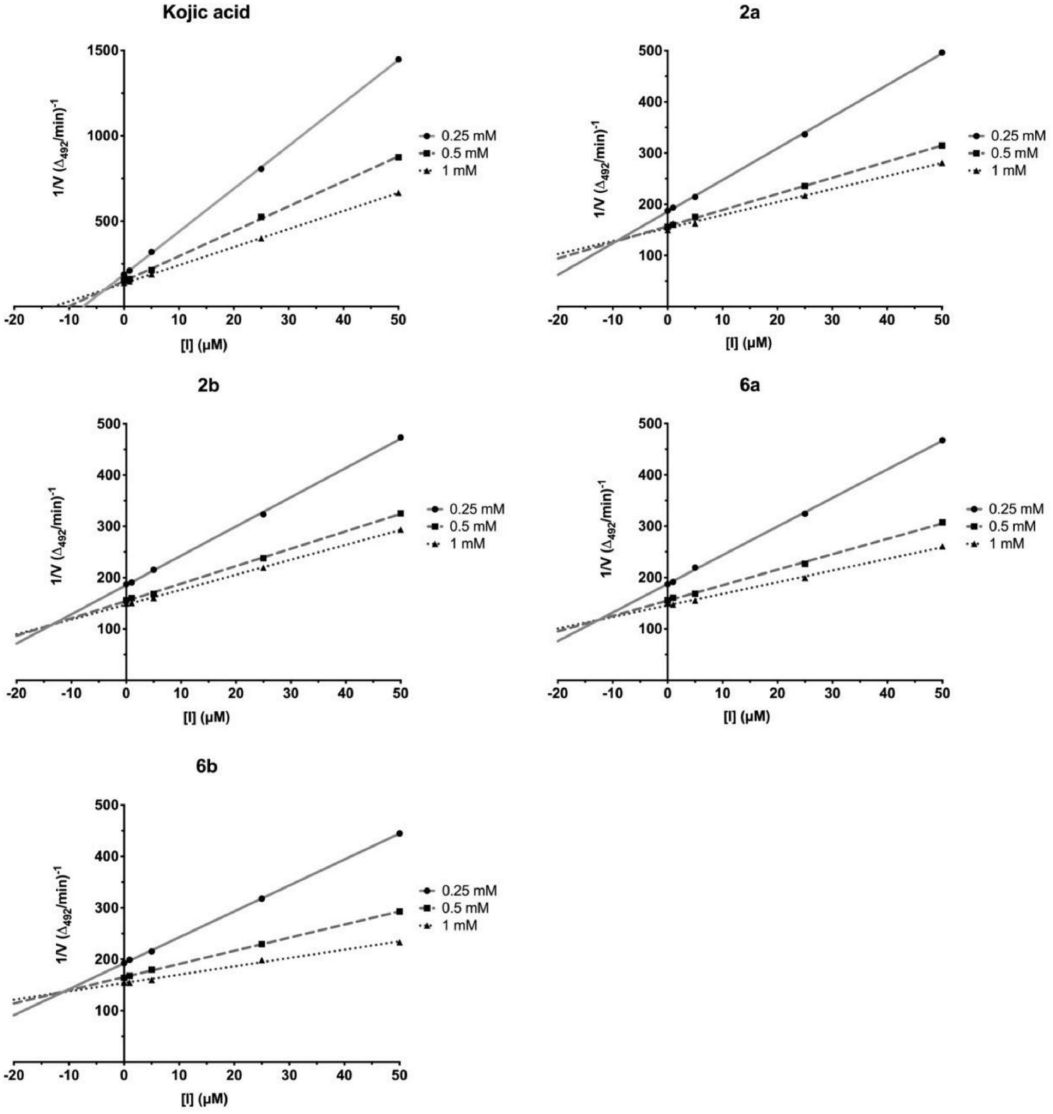
Dixon plots with varying concentrations of compounds **2a**, **2b**, **6a**, **6b**, and **KA**. Concentration of inhibitor varied from 0 to 50 μM. Three concentrations of L-tyrosine as a substrate at 0.25, 0.5, and 1 mM were used. [I]: the concentration of the inhibitor. Each point represents the combined averages of three independent experiments in triplicate under the same conditions.

**Figure 8.**
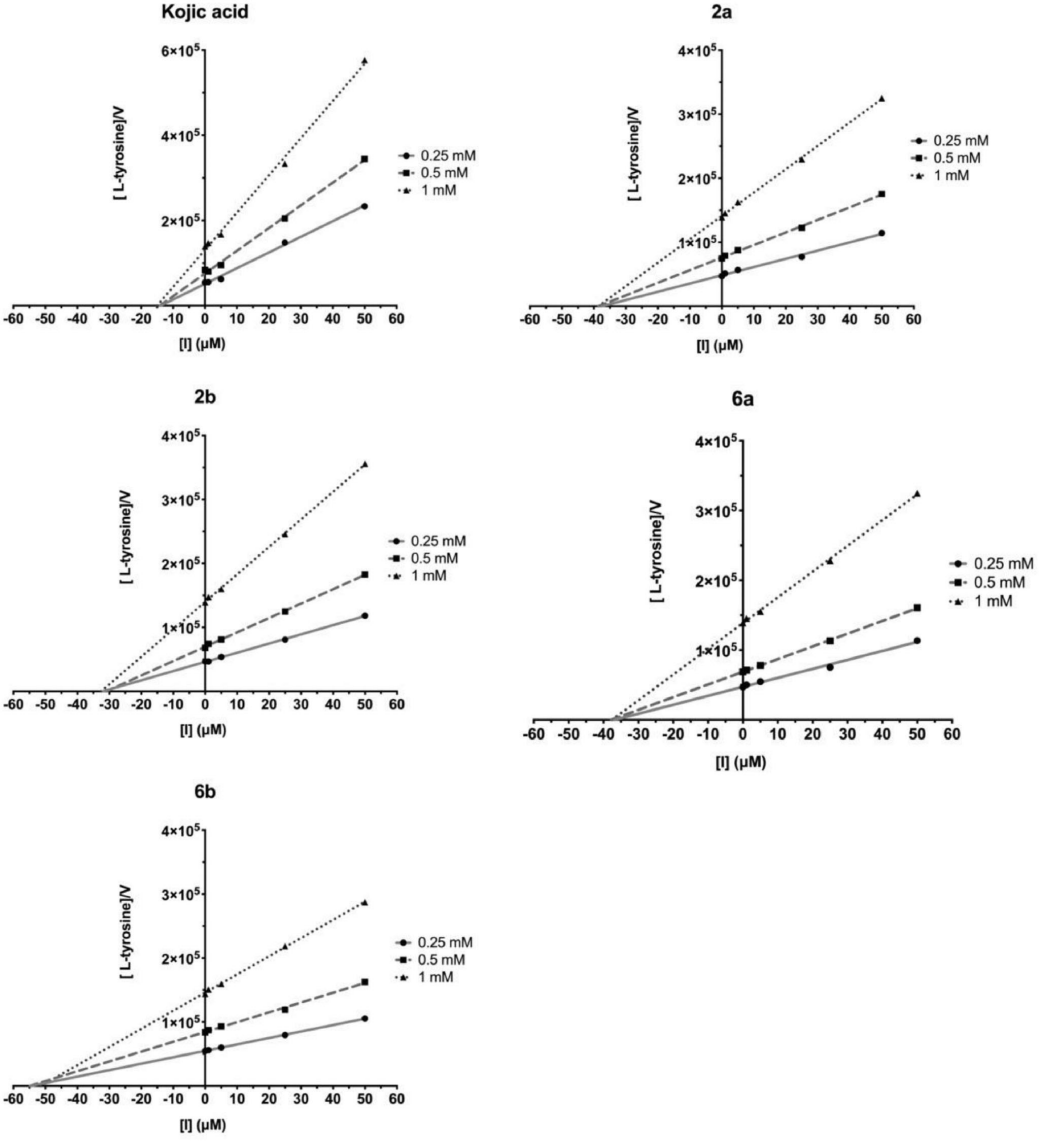
Plots of [L-tyrosine]/V *vs*. [I] with varying concentrations of compounds **2a**, **2b**, **6a**, **6b**, and **KA**. Concentration of inhibitor varied from 0 to 50 μM. Three concentrations of L-tyrosine as a substrate at 0.25, 0.5, and 1 mM were used. Each point represents the mean of triplicate experiments.

The screening studies of Tyr inhibition highlighted four distinctive thiosemicarbazides (**2a**, **2b**, **6a**, and **6b**) with significant inhibitory activity. Therefore, these compounds were subsequently subjected to detailed kinetic studies to evaluate inhibition parameters. The Lineweaver–Burk plots (Figure 6), which are the linearised transformations of the Michaelis–Menten curves, clearly demonstrate mixed character of inhibition of studied compounds. Hence, compounds **2a**, **2b**, **6a**, and **6b** cannot only bind to the enzyme at its active site and prevent the substrate from binding, but also at the allosteric site. This possibility has been addressed by docking studies (see below). From the line corresponding to the uncatalysed reaction, the values of kinetic parameters; pseudo-first-order reaction rate, V_max_/K_M_, equal to 0.0583 min^−1^ and rate of saturated enzyme, V_max_, 7.8 µM/min has been evaluated. Dixon plots (Figure 7) were used in evaluating the K_I_ values^49^, while the plots of [L-tyrosine]/V *vs.* [I] (Figure 8), were employed to determine IC_50Tyr_^50^. [I] represents the concentration of the inhibitor. Rates were monitored spectrophotometrically by detecting changes in absorbance at 492 nm. The results are collectively summarised in Table 2 and highlight the trend in inhibitory potency; *para*-nitro **2b** (IC_50Tyr_ 31.9 µM) > *meta*-iodo **6a** (IC_50Tyr_ 37.4 µM) > *para*-nitro **2a** (IC_50Tyr_ 38.3 µM) > *meta*-iodo **6b** (IC_50Tyr_ 53.6 µM), that is fortunately not linked directly with cytotoxic activity. Indeed, the most cytotoxic **6b** (CC_30_ = 117.68 µM) is the weakest inhibitor, whereas compounds **2a** and **6a** with comparable inhibitory potency differ substantially in their cytotoxicity, CC_30_ = 682.98 and 213.27 µM, respectively.

**Table 2.** Kinetic parameters of Tyr inhibition assay of compounds **2a**, **2b**, **6a** and **6b,** and **KA**.

Inhibitor	V_max_/K_M_^a^	K_M_^b^	K_I_^c^	IC_50_*_Tyr_*^d^	CC_30_^e^
None	0.0583	0.219	–	–	–
**2a**	0.0401	0.338	9.35	38.3	683.0
**2b**	0.0339	0.380	13.4	31.9	378.4
**6a**	0.0376	0.359	12.4	37.4	213.3
**6b**	0.0316	0.348	10.9	53.6	117.3
**KA**	0.0185	0.489	3.84	14.4	1558.0

^a^V_max_/K_M_ [min.^–1^] represents the catalytic efficiency as a ratio of the maximum rate of reaction (V_max_) to the Michaelis constant (K_m_).

^b^K_M_ [mM] represents Michaelis–Menten constant, the substrate concentration at which the reaction rate was 50% of the maximum rate of reaction when all enzyme active sites were saturated with substrate (V_max_).

^c^K_I_ [µM] represents the inhibitory constant, the compound concentration required to produce half maximum inhibition.

^d^IC_50_*_Tyr_* [µM] represents the concentration of the compound required for 50% inhibition of Tyr.

^e^CC_30_ [µM] represents the non-cytotoxic concentration of the compound required for 30% of cells proliferation inhibition *in vitro*.

Two scenarios of the molecular mechanism of Tyr inhibition can be proposed from our kinetic studies. Since mixed type of inhibition has been determined quite frequently for this enzyme^51^, it is apparent that the inhibitor can compete with the substrate for binding space within the enzyme active site, i.e. a pocket around the iron atom, ligated by six histidines. However, two possible structural options can be considered for the other component of the inhibition, originating from the E·S·I ternary complex. The first involves the simultaneous binding of the reactant and inhibitor to the active site of the enzyme. The second consists of the inhibitor binding to an allosteric site. Detailed docking simulations excluded the first possibility on the basis of failure of binding the substrate to the enzyme-inhibitor and of biding inhibitor to enzyme-substrate complexes within the active site. This result is not surprising since the active site is quite tight and buried. Thus, only the second option remains, which is allosteric inhibition at a remote site with unknown mechanism of action or near the active site most likely with steric hindrance preventing substrate from entering/exiting while the inhibitor is bound to the protein.

**Figure 9.**
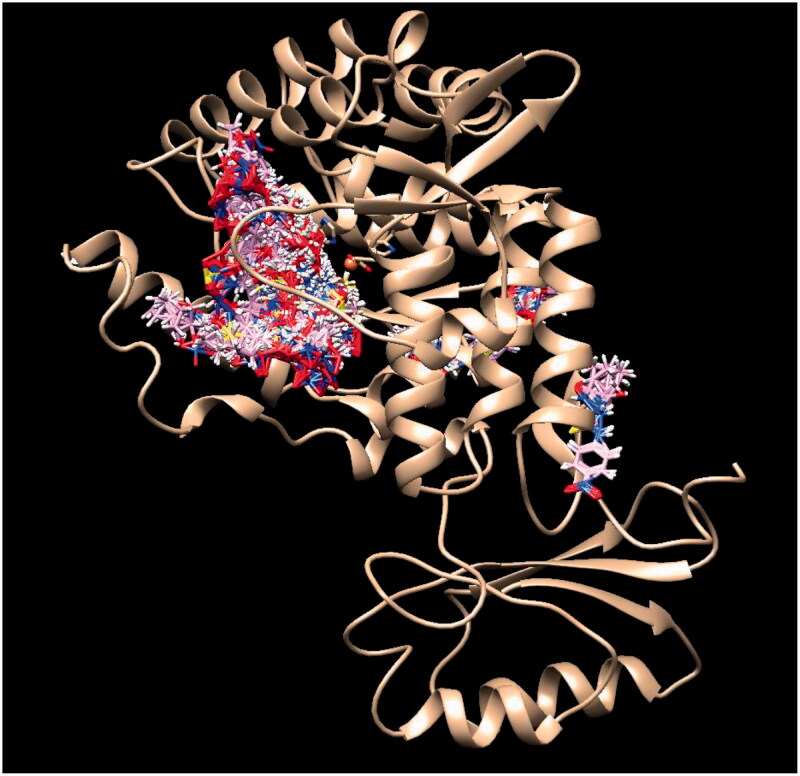
Clusters of compound **2b** poses docked to AaaH1.

Assuming that the mechanisms of inhibition of *T. gondii* TyrH (the enzyme in which catalytic mechanism we are mainly interested) and Tyr are similar, we have used the latter as a proxy. This assumption is supported by the similarity of action and the fact that mixed inhibition of allosteric origin has been reported for this class of enzymes despite of structural differences^52^^,^^53^. To explore the binding to allosteric sites, docking of the most potent inhibitor compound **2b** to AaaH1 surface rather than the active site was conducted. Docking poses clustered around three possible allosteric binding sites that are illustrated in Figure 9. The first (leftmost in the figure) is close to the entrance of the active site, which for TyrH is quite accessible. The second and third are neighbouring sites on the other side of the active site and in enzyme part analogous to that of the recently documented allosteric site in tryptophan hydroxylase^54^.

**Scheme 3.**
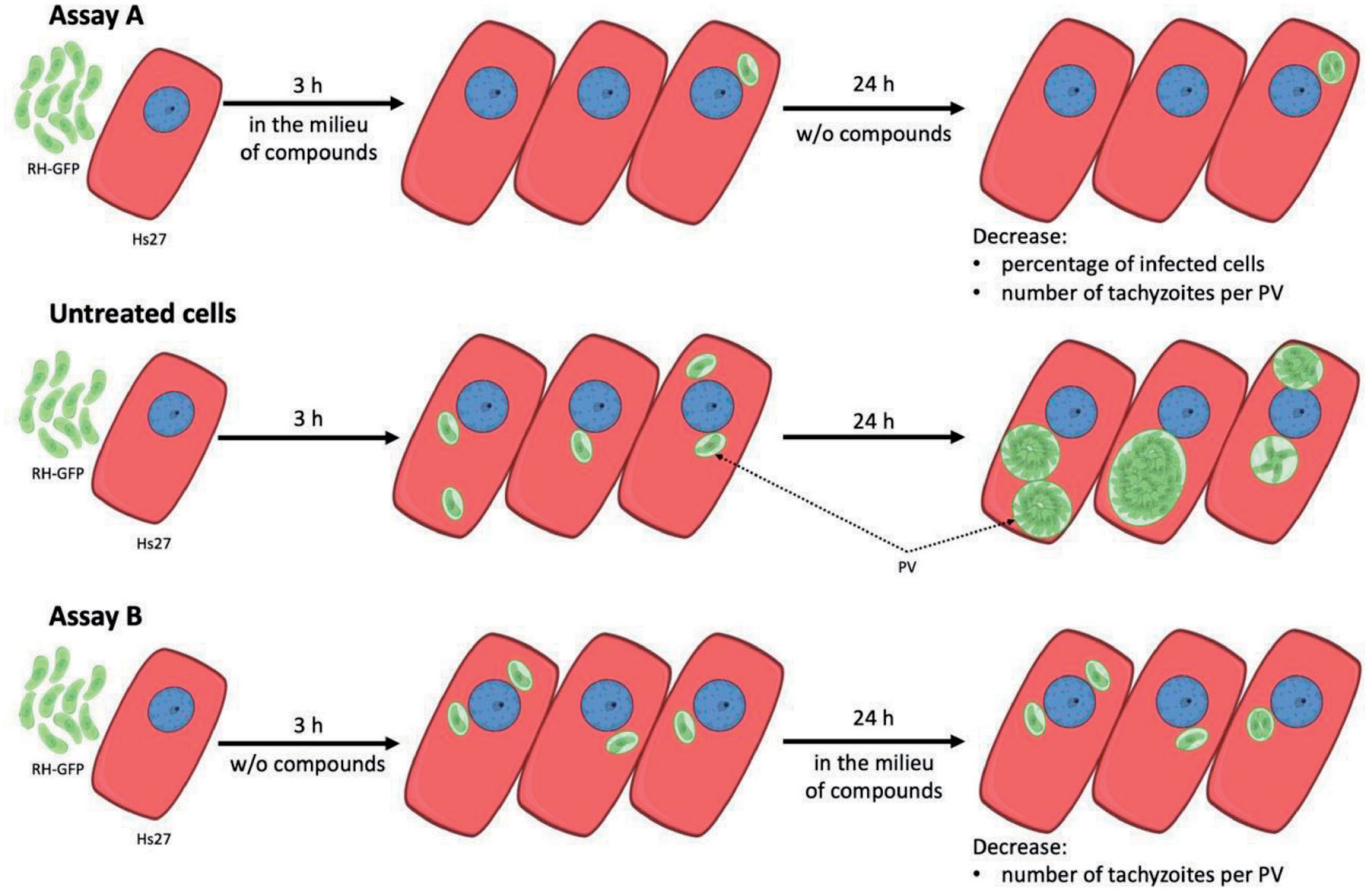
Influence of compounds during *Toxoplasma* growth. **Assay A** – compounds were present only at the time of penetration of *T. gondii* into the cells. In comparison to untreated cells, decrease in the percentage of infected cells and number of tachyzoites per PV, were observed. **Assay B** – compounds were present only for 24 h, after 3 h of *T. gondii* penetration into the cells. In comparison to untreated cells, no significant change in the percentage of infected cells but decrease in the number of tachyzoites per PV, were observed. RH-GFP: *T. gondii* tachyzoites RH strain with GFP; Hs27: human foreskin fibroblast cells; PV: parasitophorous vacuole, w/o: without.

To confirm the concept that thiosemicarbazides may act as inhibitors of *T. gondii* TyrH involved in L-tyrosine metabolism, we examined the influence of the most potent anti-Tyr compounds (**2a**, **2b**, **6a**, and **6b**) during *T. gondii* growth into human foreskin fibroblast cells. In these studies, the reference Tyr inhibitor, KA, was also included. In the first variant of the experiment (assay A), the effect of the compounds towards penetration of tachyzoites into the host cells was determined. Therefore, Hs27 cells with extracellular *T. gondii* tachyzoites were incubated for 3 h in the milieu of tested compounds or KA and then washed out. Next, infected cells were incubated further 24 h in normal medium. In this assay, the compounds were present only during penetration of *T. gondii* into the cells (Scheme 3). The confocal scanning laser microscope analyses of the host cells after assay A demonstrates that PV formation can still occur, but morphologically are smaller in comparison to untreated cells (Figure 10(A)).

Figure 10.(A) Representative images of confocal microscopy 3D parasitophorous vacuole structure imaging from assay A. Host cell infected with tachyzoites for 3 h in the milieu of compounds **2a**, **2b**, **6a**, **6b**, or **KA** and then washed, incubated for 24 h in free medium; *T. gondii* – green; NT: untreated, KA: kojic acid. (B) Representative images from assay A. Host cell infected with tachyzoites for 3 h in the milieu of compounds **2a**, **2b**, **6a**, **6b**, or **KA** and then washed, incubated for 24 h in free medium; *T. gondii* – green, cell – red, cell nuclei – blue; NT: untreated, KA: kojic acid.

**Figure:**
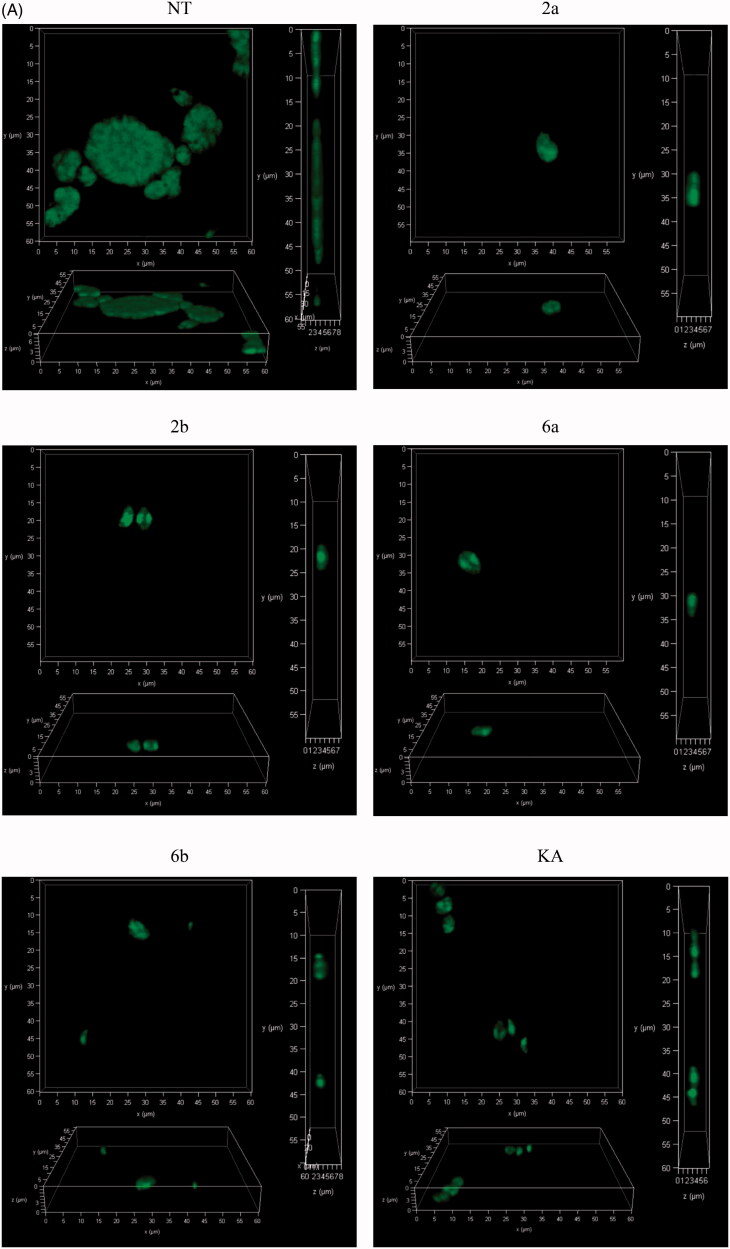

**Figure:**
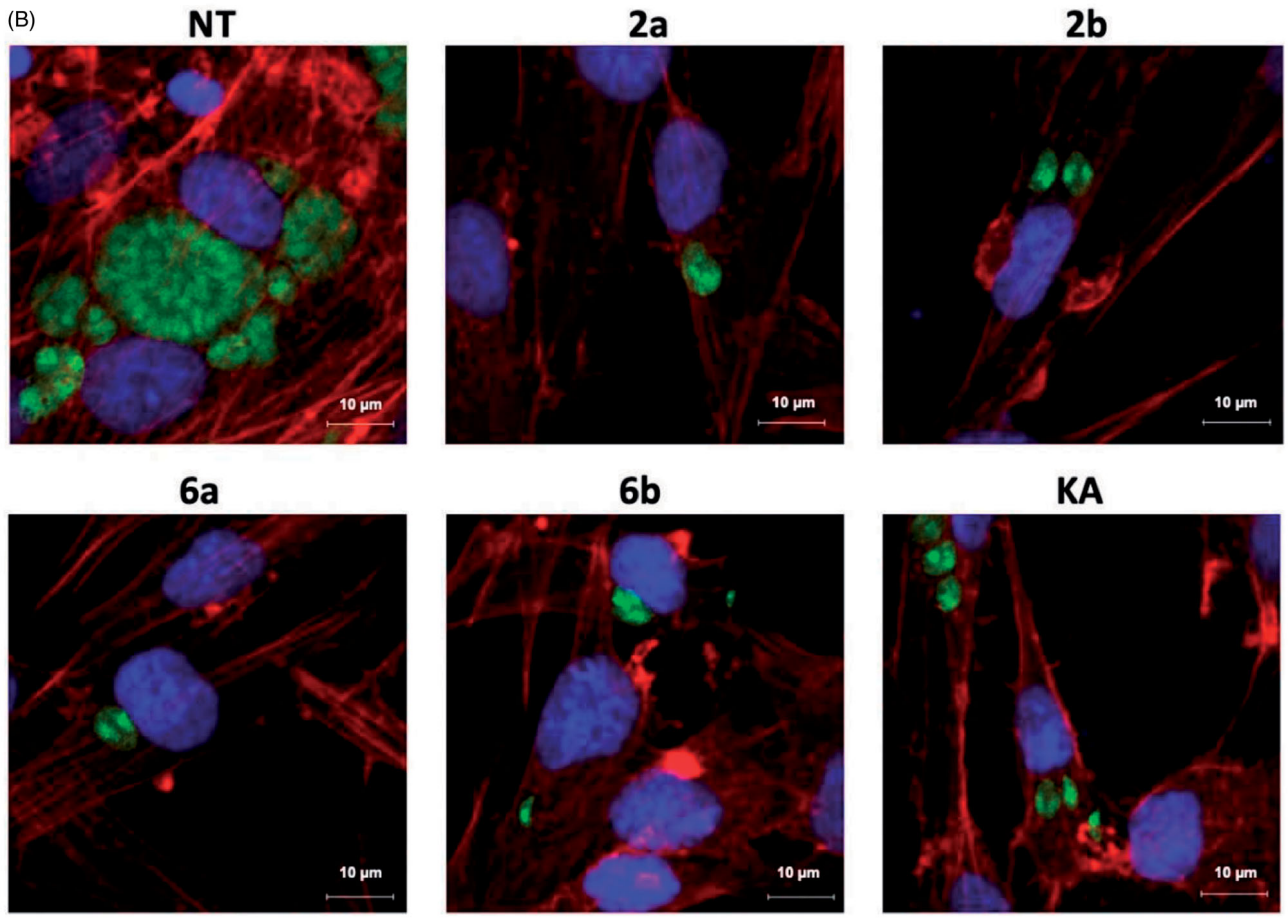

**Figure 11.**
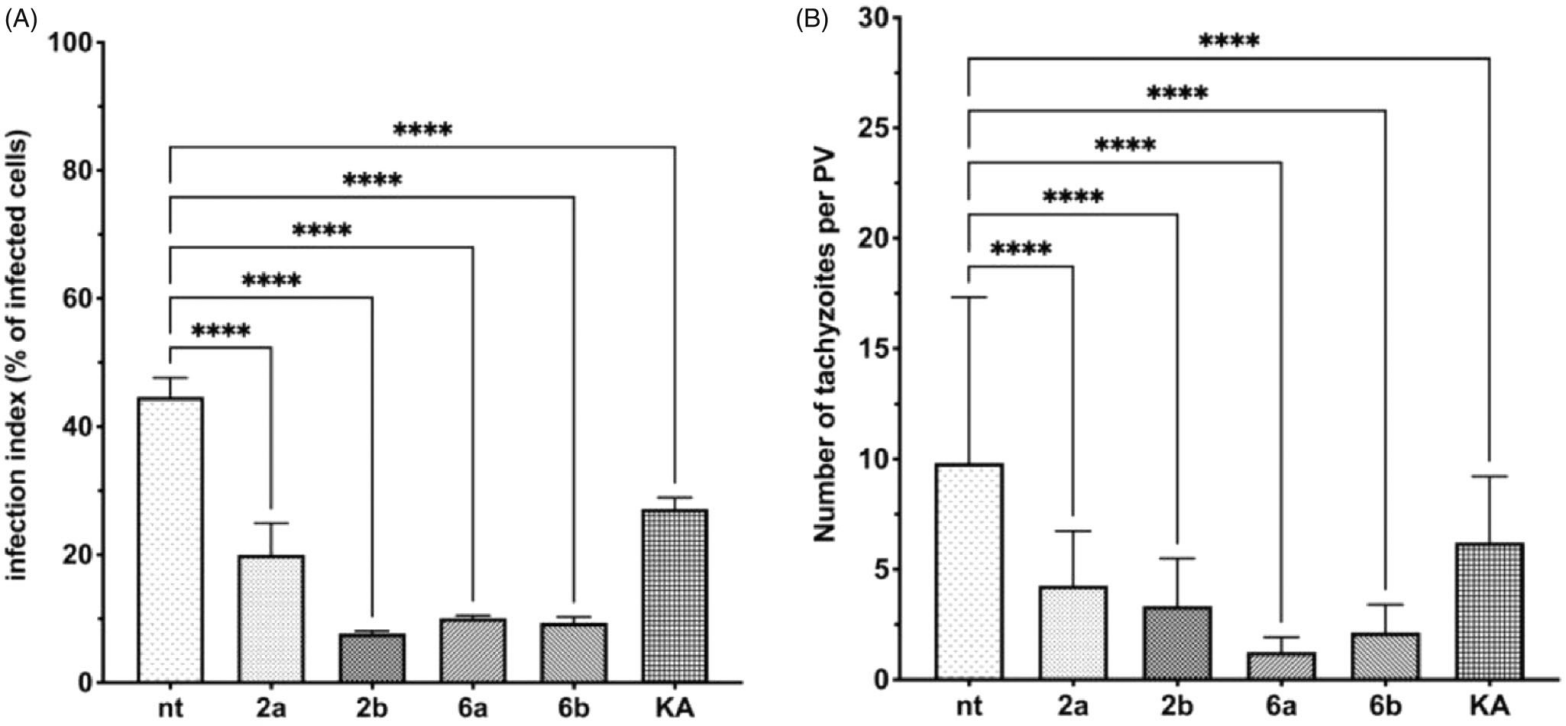
Influence of compounds during Toxoplasma growth assay A – statistical analyses. Host cell infected with tachyzoites for 3 h in the milieu of compounds **2a, 2b, 6a, 6b**, or **KA** and then washed, incubated for 24 h in free medium. (A) Infection index, percentage of infected cells. (B) Number of tachyzoites per parasitophorous vacuole. nt: untreated; KA: kojic acid; PV: parasitophorous vacuole. Data shown are the combined averages of three independent experiments on triplicate chamber slides under the same conditions. Error bars indicate standard error of the mean. Values with statistically significant differences are labelled by brackets and asterisks as follows: *p < .05; **p < .01; ***p < .001; ****p < .0001. Data were compared using a two-way ANOVA.

In the next step, the obtained data from microscopic analysis (Figure 10(B)) of assay A with selected compounds **2a**, **2b**, **6a**, **6b,** and KA, were analysed by the two-way ANOVA test and the results are summarised in Figure 11. These studied compounds effectively inhibited tachyzoites penetration into host cells in comparison to untreated cells. This effect is observed by the significant reduction in the number of infected cells (Figure 11(A)) and tachyzoites per PV (KA > **2a** = **2b** > **6b** > **6a**; Figure 11(B)) in comparison to untreated cells.

Additionally, we performed an alternative version to that described above (assay B), in which tachyzoites had 3 h to penetrate the host cells in the absence of compounds **2a**, **2b**, **6a**, **6b,** or KA. Then, the tested compounds were added and further incubated for 24 h, thus, were present only during the growth of intercellular *T. gondii* (Scheme 3). All tested Tyr inhibitors (**2a**, **2b**, **6a,** and **6b**) as well as reference KA, similar to assay A, had an effect on the size of the formed PVs compared to untreated cells (Figure 12(A)).

Figure 12.(A) Representative images of confocal microscopy 3D parasitophorous vacuole structure imaging from assay B. Impeding tachyzoites division in host cell after 24 h incubation with tested compounds or KA; *T. gondii* – green; NT: untreated, KA: kojic acid. (B). Representative images from assay B. Impeding tachyzoites division in host cell after 24 h incubation with tested compounds or KA; *T. gondii* – green, cell – red, cell nuclei – blue; NT: untreated; KA: kojic acid.

**Figure:**
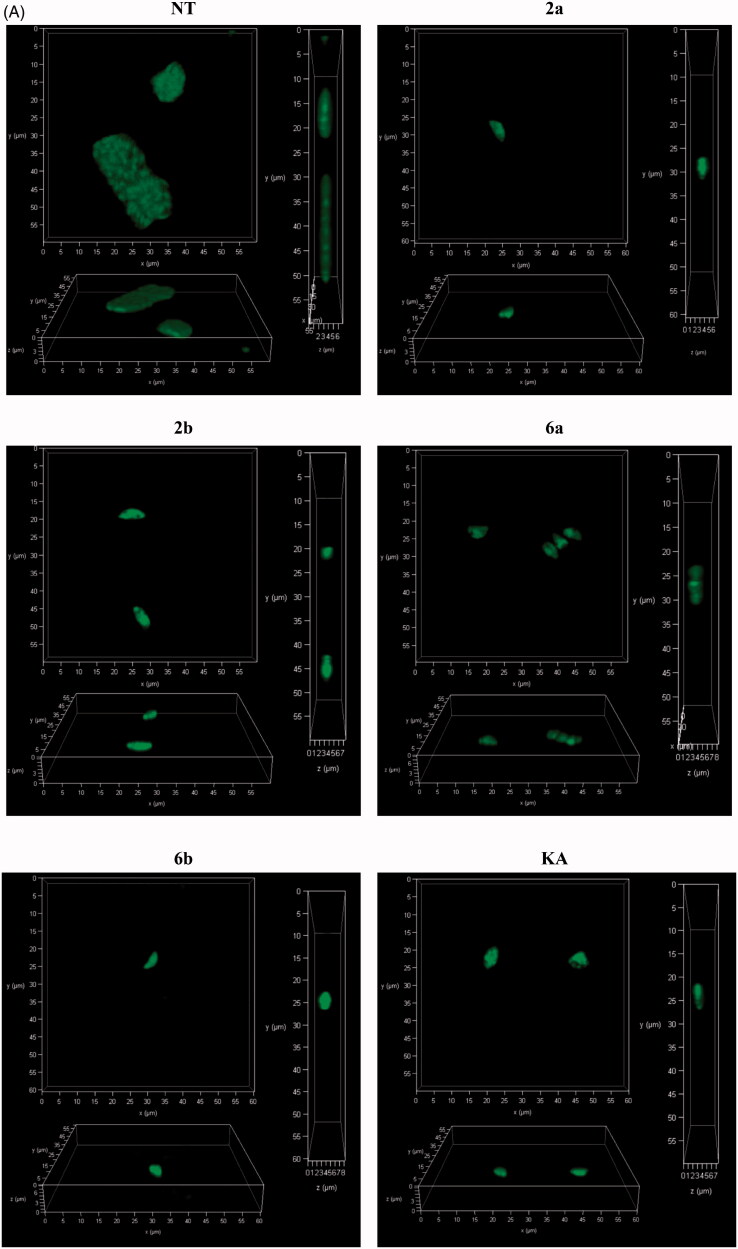

**Figure:**
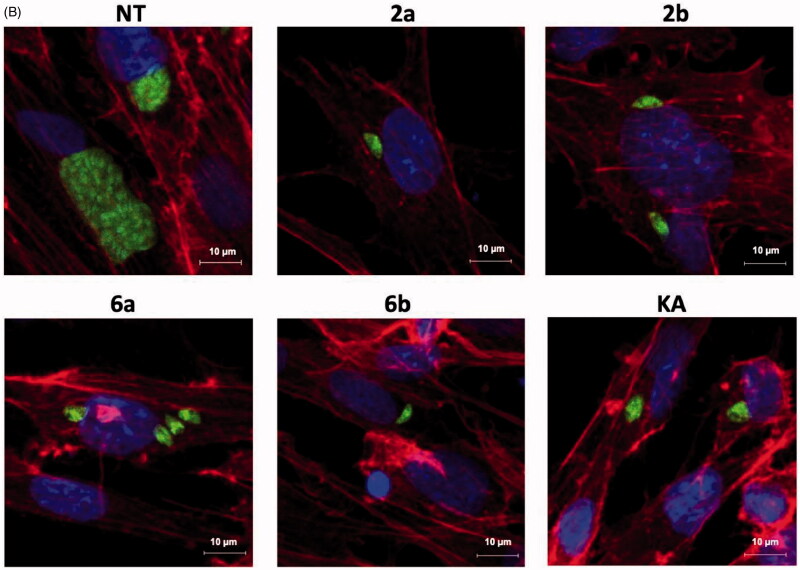

**Figure 13.**
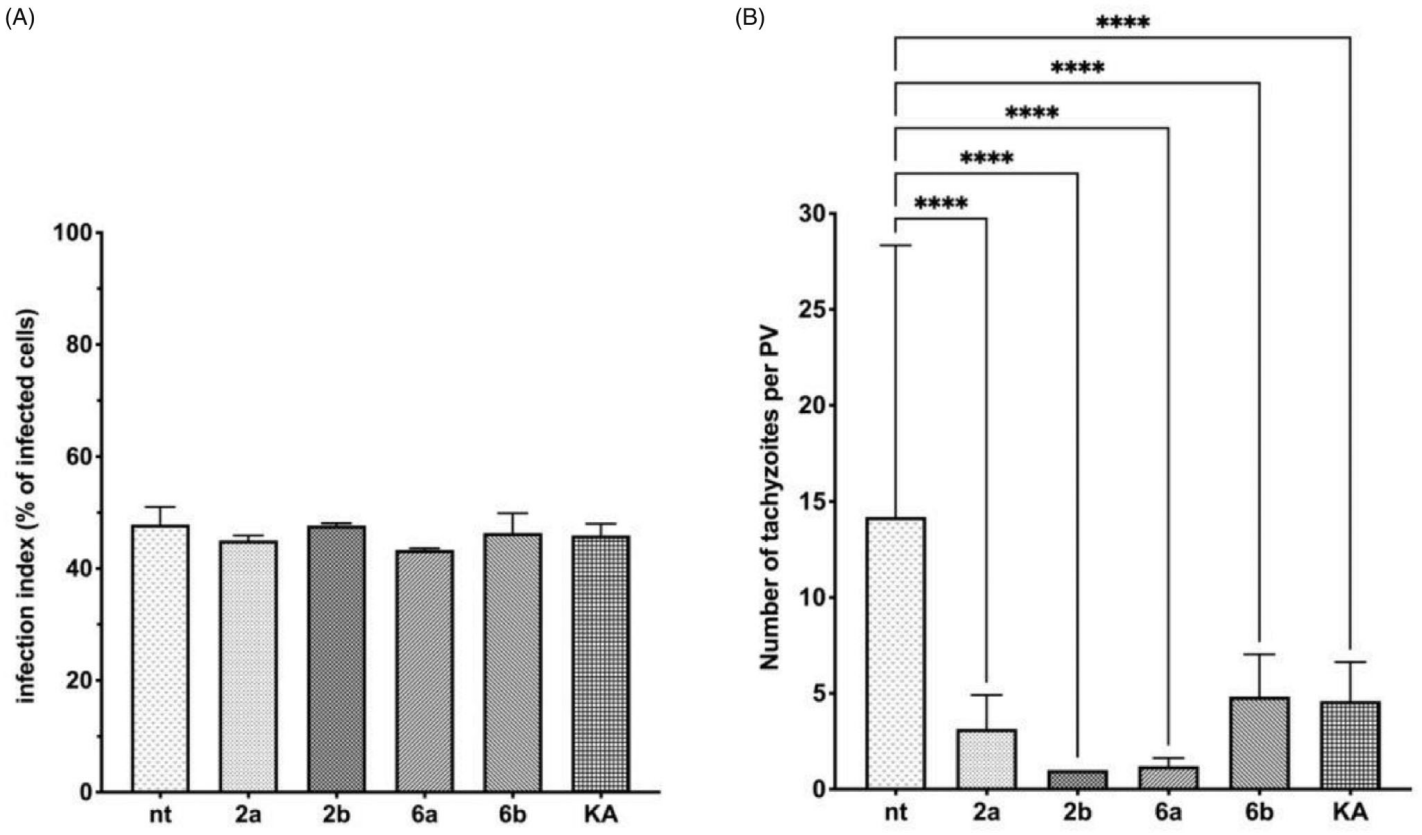
Influence of compounds during *Toxoplasma* growth assay B – statistical analyses. Host cell infected with tachyzoites for 3 h in free medium, then washed and incubated 24 h in the milieu of compounds **2a**, **2b**, **6a**, **6b**, or **KA**. (A) Infection index, percentage of infected cells. (B) Number of tachyzoites per parasitophorous vacuole. nt: not treated; KA: kojic acid; PV: parasitophorous vacuole. Data shown are the combined averages of three independent experiments on triplicate chamber slides under the same conditions. Error bars indicate standard error of the mean. Values with statistically significant differences are labelled by brackets and asterisks as follows: *p < .05; **p < .01; ***p < .001; ****p < .0001. Data were compared using a two-way ANOVA.

The results from microscopic analysis of assay B (Figure 12(B)) are summarised in Figure 13, and were analysed by the two-way ANOVA test. As expected, the difference in percentage of infected cells is not observable, in comparison to untreated cells, due to in assay B *T. gondii* has time (3 h without compounds) to enter the cell and for initial PV formation (Figure 13(A)). However, the significant reduction in the number of tachyzoites per PV is visible (Figure 13(B)). Compounds **2a**, **2b**, **6a**, and **6b** significantly inhibit intracellular parasite proliferation by impeding tachyzoites division (KA = **6b** > **2a** > **6a** = **2b**) in comparison to untreated cells.

Recently, Marino and Boothroyd^19^ observed a decrease in the number of PV per cell in L-tyrosine-free medium as well as the average number of doublings tachyzoites post-infection, which is related to *Toxoplasma* tachyzoites requiring exogenous L-tyrosine for growth. The presence of the studied compounds can disrupt L-tyrosine metabolism in tachyzoites. In both situations, a significant decrease in the number of tachyzoites per PV is observed (Scheme 3).

Conclusively, the results obtained for *in silico* predictions of molecular basis of anti-*T. gondii* activity of thiosemicarbazide-based compounds combined with experimental influence of compounds during *Toxoplasma* growth (assay A and B) support conclusion that the studied compounds are able to disrupt L-tyrosine metabolism in tachyzoites by deregulation of TyrH. In order to further provide evidence for this proposition, enzymatic experiments should be conducted. An alternative explanation may be that thiosemicarbazide-based compounds target the plasma membrane-localised amino acid transporter(s), which is important for L-tyrosine uptake into parasite. This hypothesis originates from the studies of Marino and Boothroyd^19^ that recently confirmed that *Toxoplasma* tachyzoites are auxotrophic for exogenous L-tyrosine, which must be salvaged from their host cells *via* direct uptake or degradation of host proteins. In addition, Wang et al.^55^ reported that although recombinant AaaH1 and AaaH2 are capable of converting phenylalanine to L-tyrosine, in which their expression levels during tachyzoite growth are exceedingly low. These reports highlight that L-tyrosine transporter localised to the plasma membrane of tachyzoites might be an alternative key target for anti-*Toxoplasma* activity of thiosemicarbazide-based compounds. This hypothesis is beyond the scope of this study and will be considered in our future work.

## Materials and methods

### Chemistry

The prepared compounds were characterised using ^1^H and ^13 ^C NMR spectroscopy. Each tested compound had ≥96% purity, as determined using elemental analysis. All chemicals used were obtained from Sigma-Aldrich (Saint Louis, MO) and Alfa Aesar (Karlsruhe, Germany) and used without further purification. TLC separations were performed on Merck aluminium plates with silica gel 60 F_254_ (Darmstadt, Germany). Melting points were determined using a Fischer-Johns block and are uncorrected. Elemental analyses were determined by an AMZ-CHX elemental analyser (PG, Gdansk, Poland), and all determined elements were within ±0.4% of the theoretical values. ^1^H and ^13 ^C NMR spectra were recorded using a Bruker Avance spectrometer (Bruker BioSpin GmbH, Rheinstetten, Germany) using DMSO-d_6_ as solvent. Chemical shifts are reported as *δ* values in parts per million (ppm) and indirectly referenced to tetramethylsilane *via* the solvent signal. Physicochemical characterisations of compounds **1a**–**9a** were described previously^34^^,^^35^. The structures of compounds **1b**–**3b, 7b,** and **8b** are known (CAS numbers: 891079–50-8, 891079–58-6, 891075–55-1, 891069–84-4, 891062–80-9, respectively), however, there are no references reporting their use, preparation or physicochemical characterisation. Therefore, this data is included.

### General method for the synthesis of thiosemicarbazides 1a–9a and 1b–9b

The corresponding aryl isothiocyanate (0.01 mol) was added to a solution of 4-methylimidazole-5-carbohydrazide (0.01 mol) or cyclopentanecarbohydrazide (0.01 mol) in anhydrous ethanol (25 mL). The reaction mixture was heated under reflux until the isothiocyanate agent was consumed as determined by TLC (mobile phase: CHCl_3_/EtOH, 3:1, 2:1, or 1:1). The final product was filtered from the reaction mixture and purified by crystallisation from EtOH.

#### 1-Cyclopentanoyl-4–(3-nitrophenyl)thiosemicarbazide (1b)

Yield: 74% (yellowish solid); mp 134–136 °C. R*f* value: 0.24. ^1^H NMR (DMSO-d_6_): δ 1.55–1.83 (m, 8H, 4 × CH_2_), 2.67–2.72 (m, 1H, 1 × CH), 7.60–7.64 (m, 1H, 1 × CH_ar_), 8.00 (s, 2H, 2 × CH_ar_), 8.50 (m, 1H, 1 × CH_ar_), 9.87 (s, 1H, 1 × NH), 9.90 (2 s, 2H, 2 × NH). ^13 ^C NMR (DMSO-d_6_): δ 181.33, 176.01, 147.68, 141.00, 132.10, 129.65, 124.61, 120.03, 42.83, 30.39, 26.14. Anal. Calcd for C_13_H_16_N_4_O_3_S: C, 50.64; H, 5.23; N, 18.17. Found: C, 50.33; H, 5.27; N, 18.22.

#### 1-Cyclopentanoyl-4–(4-nitrophenyl)thiosemicarbazide (2b)

Yield: 67% (yellowish solid); mp 170–172 °C. R*f* value: 0.10. ^1^H NMR (DMSO-d_6_): δ 1.54–1.83 (m, 8H, 4 × CH_2_), 2.67–2.72 (m, 1H, 1 × CH), 7.88–7.92 (dd, 2H, 2 × CH_ar_), 8.20–8.23 (dd, 2H, 2 × CH_ar_), 9.91 (s, 2H, 2 × NH), 9.98 (s, 1H, 1 × NH). ^13 ^C NMR (DMSO-d_6_): δ 180.98, 176.02, 146.15, 143.77, 124.99, 124.12, 42.47, 30.38, 26.19. Anal. Calcd for C_13_H_16_N_4_O_3_S: C, 50.64; H, 5.23; N, 18.17. Found: C, 50.59; H, 5.52; N, 18.18.

#### 4–(2-Chlorophenyl)-1-cyclopentanoylthiosemicarbazide (3b)

Yield: 52% (white solid); mp 178–180 °C. R*f* value: 0.89. ^1^H NMR (DMSO-d_6_): δ 1.54–1.82 (m, 8H, 4 × CH_2_), 2.64–2.67 (m, 1H, 1 × CH), 7.26–7.34 (m, 2H, 2 × CH_ar_), 7.48–7.51 (m, 2H, 2 × CH_ar_), 9.32; 9.71; 9.93 (3 s, 3H, 3 × NH). ^13 ^C NMR (DMSO-d_6_): δ 181.88, 175.97, 137.00, 131.05, 130.60, 129.73, 128.13, 127.51, 42.76, 30.23, 26.15. Anal. Calcd for C_13_H_16_ClN_3_OS: C, 52.43; H, 5.42; N, 14.11. Found: C, 52.72; H, 5.63; N, 14.28.

#### 4–(2-Bromophenyl)-1-cyclopentanoylthiosemicarbazide (4b)

Yield: 85% (white solid); mp 179–181 °C. R*f* value: 0.68. ^1^H NMR (DMSO-d_6_): δ 1.54–1.82 (m, 8H, 4 × CH_2_), 2.62–2.70 (m, 1H, 1 × CH), 7.16–7.21 (m, 1H, 1 × CH_ar_), 7.35–7.41 (m, 1H, 1 × CH_ar_), 7.55–7.57 (m, 1H, 1 × CH_ar_), 7.64–7.67 (m, 1H, 1 × CH_ar_), 9.31, 9.70, 9.93 (3 s, 3H, 3 × NH). ^13 ^C NMR (DMSO-d_6_): δ 181.72, 176.03, 138.38, 132.86, 130.65, 128.43, 128.14, 121.91, 42.76, 30.25, 26.17. Anal. Calcd for C_13_H_16_BrN_3_OS: C, 45.62; H, 4.71; N, 12.28. Found: C, 45.51; H, 4.88; N, 12.06.

#### 1-Cyclopentanoyl-4–(2-iodophenyl)thiosemicarbazide (5b)

Yield: 67% (white solid); mp 182–184 °C. R*f* value: 0.57. ^1^H NMR (DMSO-d_6_): δ 1.51–1.80 (m, 8H, 4 × CH_2_), 2.62–2.70 (m, 1H, 1 × CH), 6.99–7.04 (m, 1H, 1 × CH_ar_), 7.37–7.48 (m, 2H, 2 × CH_ar_), 7.86–7.88 (d, 1H, 1 × CH_ar_), 9.28, 9.65, 9.92 (3 s, 3H, 3 × NH). ^13 ^C NMR (DMSO-d_6_): δ 175.99, 171.78, 149.64, 131.20, 129.00, 126.81, 122.37, 118.34, 42.50, 30.25, 26.19. Anal. Calcd for C_13_H_16_IN_3_OS: C, 40.11; H, 4.14; N, 10.80. Found: C, 39.97; H, 4.04; N, 10.85.

#### 1-Cyclopentanoyl-4–(3-iodophenyl)thiosemicarbazide (6b)

Yield: 67% (white solid); mp 173–175 °C. R*f* value: 0.58. ^1^H NMR (DMSO-d_6_): δ 1.57–1.82 (m, 8H, 4 × CH_2_), 2.61–2.72 (m, 1H, 1 × CH), 7.11–7.16 (m, 1H, 1 × CH_ar_), 7.50–7.53 (m, 2H, 2 × CH_ar_), 7.84–7.89 (m, 1H, 1 × CH_ar_), 9.60, 9.66, 9.83 (3 s, 3H, 3 × NH). ^13 ^C NMR (DMSO-d_6_): δ 181.17, 175.86, 141.13, 133.89, 132.29, 130.91, 130.42, 125.56, 42.71, 30.17, 26.15. Anal. Calcd for C_13_H_16_IN_3_OS: C, 40.11; H, 4.14; N, 10.80. Found: C, 40.36; H, 4.22; N, 10.87.

#### 1-Cyclopentanoyl-4–(2-methoxyphenyl)thiosemicarbazide (7b)

Yield: 86% (white solid); mp 176–178 °C. R*f* value: 0.76. ^1^H NMR (DMSO-d_6_): δ 1.54–1.82 (m, 8H, 4 × CH_2_), 2.66–2.69 (m, 1H, 1× CH), 3.81 (s, 3H, OCH_3_), 6.93–7.04 (m, 1H, 1 × CH_ar_), 7.06–7.07 (m, 1H, 1 × CH_ar_), 7.11–7.14 (m, 1H, 1 × CH_ar_), 8.31–8.33 (m, 1H, 1 × CH_ar_), 8.87, 9.70, 10.00 (3 s, 3H, 3 × NH). ^13 ^C NMR (DMSO-d_6_): δ 180.19, 176.12, 151.16, 128.09, 125.78, 123.85, 120.26, 111.63, 56.50, 42.61, 30.55, 26.19. Anal. Calcd for C_14_H_19_N_3_O_2_S: C, 57.31; H, 6.53; N, 14.32. Found: C, 57.55; H, 6.48; N, 14.21.

#### 1-Cyclopentanoyl-4–(4-methylphenyl)thiosemicarbazide (8b)

Yield: 54% (white solid); mp 175–177 °C. R*f* value: 0.84. ^1^H NMR (DMSO-d_6_): δ 1.56–1.81 (m, 8H, 4 × CH_2_), 2.28 (s, 3H, CH_3_), 2.61–2.69 (m, 1H, 1 × CH), 7.12–7.14 (dd, 2H, 2× CH_ar_), 7.28–7.31 (dd, 2H, 2 ×CH_ar_), 9.45 (s, 2H, 2 × NH), 9.80 (s, 1H, 1 × NH). ^13 ^C NMR (DMSO-d_6_): δ 181.48, 175.69, 137.05, 134.57, 129.01, 126.18, 42.74, 30.18, 26.22, 21.03. Anal. Calcd for C_14_H_19_N_3_OS: C, 60.62; H, 6.90; N, 15.15. Found: C, 60.53; H, 7.07; N, 15.17.

#### 1-Cyclopentanoyl-4–(2,5-difluorophenyl)thiosemicarbazide (9b)

Yield: 56% (white solid); mp 178–180 °C. R*f* value: 0.87. ^1^H NMR (DMSO-*d_6_*): δ 1.54–1.79 (m, 8H, 4 × CH_2_), 2.63–2.68 (m, 1H, 1 ×CH), 7.15 (s, 1H, 1 × CH_ar_), 7.27–7.35 (m, 2H, 2 × CH_ar_), 9.35, 9.84, 9.92 (3 s, 3H, 3 × NH). ^13 ^C NMR (DMSO-d_6_): δ 182.07, 174.93, 159.19, 128.72, 117.22, 117.09, 116.92, 116.79, 42.46, 30.18, 26.14. Anal. Calcd for C_13_H_15_F_2_N_3_OS: C, 52.16; H, 5.05; N, 14.04. Found: C, 51.98; H, 5.22; N, 14.21.

### Biological evaluation

All experiments were carried out in BSL-2 laboratory with biological safety cabinet. Compounds **1a**–**9a** and **1 b**–**9b** were dissolved in dimethyl sulfoxide (DMSO, Sigma-Aldrich, St. Louis, MO) to 100 mM. The final concentration of DMSO in compound dilutions was below 1.00%. TRI and PYR (92131 and 46706, Sigma-Aldrich) was dissolved in DMSO to 25 mM. The final concentration of DMSO in TRI dilution was below 1.00%. Tyr inhibitor, KA (K3125, Sigma-Aldrich), was dissolved in DMSO to 100 mM. The final concentration of DMSO in KA dilution was below 1.00%. All dilutions were freshly prepared before experiments.

#### Cell culture

The L929 mouse fibroblast (ATTC^®^ CCL-1^™^, Manassas, VA) was routinely maintained in RPMI 1640 media (R8758, Sigma-Aldrich, Suffolk, UK), supplemented with 10% foetal bovine serum (Foetal Bovine Serum, ATCC^®^ 30–2020^™^), 100 I.U./mL penicillin and 100 µg/mL streptomycin (Penicillin-Streptomycin Solution ATCC^®^ 30–2300^™^). Cells were trypsinised (Trypsin-EDTA Solution, 1X ATCC^®^ 30–2101™) twice a week and seeded at a density of 1 × 10^6^ per T25 cell culture flask and incubated at 37 °C and 10% CO_2_ to achieve a confluent monolayer.

#### Cell viability assay

Cell viability assay was performed according to international standards (ISO 10993–5:2009(E)), using tetrazolium salt (MTT, Sigma-Aldrich) and mouse fibroblasts L929 cells. Culture medium RPMI 1640, without phenol red (Biowest, Nuaille, France) supplemented with 10% FBS, 2 mM L-glutamine (Sigma-Aldrich), 100 I.U./mL penicillin, 100 µg/mL streptomycin were used. Briefly, 1 × 10^4^/well of L929 cells were placed in 96-well plates and incubated for 24 h at 37 °C and 10% CO_2_. Afterwards, the old culture medium was replaced with 100 µL of the compound, drug or inhibitor, diluted in culture medium, and the cells treated for 24 h. Additionally, cells were treated with 0.03–4.0% concentration of DMSO as the solvent (data not shown). Then, 50 µL of 1 mg/mL of MTT solution in RPMI 1640 without phenol red was added to each well and incubated for 2 h at 37 °C and 10% CO_2_. Next, cell culture medium was aspirated carefully and 150 µL of DMSO was added to each well, and the plates were gently mixed. Then, 25 µL 0.1 M glycine buffer (pH 10.5) (Sigma-Aldrich) was added. The optical density at 570 nm using a multi-mode microplate reader SpectraMax^®^ i3 (Syngen, Taipei City, Taiwan) was recorded. The results were expressed as a percentage of viability compared to untreated cells. All experiments were performed in triplicate. Cytotoxicity assay using human fibroblast Hs27 cells was also performed as described above (included in Supplementary Material).

#### Parasite culture

The Hs27 (human foreskin fibroblast) (ATCC^®^ CRL-1634^™^) was cultured in accordance with the ATCC product sheet. In short, cells were cultured in DMEM (Dulbecco’s Modified Eagle’s Medium-ATCC^®^ 30–2002^™)^ with 10% FBS, 100 I.U./mL penicillin, and 100 µg/mL streptomycin. When the cell line achieved a confluent monolayer, it was trypsinised and seeded at a density of 1 × 10^6^ per T25 cell culture flask and incubated (48–72 h, 37 °C, 5% CO_2_). The highly virulent and those belonging to the first haplogroup *Toxoplasma gondii* RH strain (ATCC^®^ PRA­310^™^) and RH-GFP (ATCC^®^ 50940^™^, expression of green fluorescent protein) strains were maintained as tachyzoites according to the ATCC product sheet, in parasite culture medium, which contains DMEM medium with 3% HIFBS (Heat-Inactivated FBS; 1 h in 56 °C). Infected tissue culture cells were incubated (37 °C, 5% CO_2_).

#### Antiparasitic assay

The influence of thiosemicarbazides **1a**–**9a**, **1b**–**9b**, TRI, PYR, and KA on *T. gondii* RH proliferation was examined using the following method: 1 × 10^4^/well of Hs27 cells were seeded on 96-well plates in cell culture medium. After 72 h of incubation, the medium was removed and then 1 × 10^5^/well tachyzoites of the RH strain were added to the cell monolayers, in parasite culture medium. After 1 h the thiosemicarbazides, drugs, or KA dilutions in the parasite culture medium were added to the Hs27 cells with *T. gondii* (concentration range of the compounds 0–500 µM, TRI 0–125 µM, and KA 0–1500 µM). After subsequent 24 h of incubation, 1 µCi/well [5,6-^3^H] uracil (Moravek Biochemicals Inc., Brea, CA) was added to each microculture for a further 72 h. The amount of isotope incorporated into the parasite nucleic acid pool, corresponded to the parasite growth, and was measured by liquid scintillation counting using 1450 Microbeta Plus Liquid Scintillation Counter (Wallac Oy, Turku, Finland). The results were expressed as counts per minute (CPM) and transformed to the percentage of viability compared to untreated cells. Finally, the inhibitory concentrations for 50% inhibition of *T. gondii* proliferation (IC_50Tg_) were calculated. All experiments were performed in triplicate.

#### Influence of compounds during toxoplasma growth (assay A and B – Scheme 3)

Hs27 cells were cultured on Lab-Tek^™^ 4-well Chamber Slides (Nunc) (5 × 10^4^ cells/500 µL per well).

**Assay A** – after 48 h, the medium was removed and then 5 × 10^5^/500 µL/well tachyzoites of the RH-GFP strain were added to the cell monolayers, in parasite culture medium with the selected compounds at IC_50Tg_ concentration: **2a**, **2b**, **6a**, **6b,** and KA, for 3 h. Then, the cells were washed to remove extracellular parasites and parasite culture medium (without any compounds) was added for 24 h.

**Assay B** – after 48 h, the medium was removed and then 5 × 10^5^/500 µL/well tachyzoites of the RH-GFP strain were added to the cell monolayers, in parasite culture medium for 3 h. Then, the cells were washed to remove extracellular parasites, and the cells were treated with the selected compounds at IC_50Tg_ concentration: **2a**, **2b**, **6a**, **6b,** and KA, for 24 h.

In both assays as the controls, Hs27 cells were infected, but not treated. After subsequent 24 h of incubation (assay A and B), the slides were washed with sterile PBS, fixed with formaldehyde solution (252549, Sigma-Aldrich), 3.7% in PBS for 20 min, and stained with DAPI for 10 min (1 mg/mL, ThermoFisher Scientific, Waltham, MA) and Texas Red^™^-X Phalloidin for 40 min (1.65 µM; ThermoFisher Scientific). Microscopic analysis of 100 cells in turn, comprised counting the infected cell ratio and tachyzoites per each PVs of infected cell, was performed using a fluorescent microscope (Olympus IX50, Tokyo, Japan) at a magnification ×1000. Additionally, cells with PVs were visualised using a confocal scanning laser microscope, Leica TCS SP8, equipped with Leica LAS AF Lite Software for image processing. Three independent experiments on triplicate chamber slides using the same conditions for both assay were performed.

#### Inhibition of tyrosinase activity assay

In our study, we used Tyr from mushrooms (T3824, Sigma) for all experiments. All compound dilutions, L-tyrosine a Tyr substrate, and Tyr were prepared in 50 mM phosphate buffer (pH 6.5). The volume of the reaction mixture was 200 µL and contained: 80 µL of 2.5 mM L-tyrosine, 50 µL of 100 µM compound/inhibitor, and 70 µL of Tyr (10 U). First, the substrate and compound or inhibitor were added to a 96-well flat-bottom plate (Nunc MaxiSorp^™^, Rosklide, Denmark), next Tyr was added and the initial absorbance (A_0_) was measured spectrophotometrically at 492 nm. Then, the plate was incubated at 25 °C for 30 min. After incubation, the amount of dopachrome produced in the reaction mixture was determined spectrophotometrically at 492 nm (A_30_) using the multi-mode microplate reader SpectraMax^®^ i3 (Syngen). 100% Tyr activity we used for the sample without addition compound/inhibitor. First, we established the inhibitory concentration of KA (IC_50KA_ = 25 µM) for 10 U of Tyr. Then, the inhibition of Tyr activity screening test was performed at 25 µM for the studied compounds. Also, IC_50_*_Tyr_* for the most active compounds; **2a**, **2b**, **6a**, and **6b** was determined. The average results from three experiments are shown.

#### Kinetic analysis of the inhibition of tyrosinase

Solutions containing 80 µL of 0.625, 1.25, or 2.5 mM L-tyrosine and 50 µL of 20, 100, or 200 µM inhibitor were added to a 96-well plate. Then, solution containing 70 µL of Tyr (10 U) was added and the initial rate of dopachrome formation was determined through detecting the increase in absorbance at the wavelength of 492 nm (Δ_492_/min). The results were linearised using Lineweaver–Burk plots from which V_max_/K_M_ and V_max_ for the reaction without the inhibitor were evaluated (K_M_ – the Michaelis–Menten constant, V_max_ – the maximum reaction velocity). In order to determine the inhibition constants K_I_ the Dixon plots were constructed, whereas the plots of [L-tyrosine]/V *vs.* inhibitor concentration [I] were used for IC_50Tyr_ evaluation. The results from three experiments are shown.

#### Docking studies

The docking calculations were performed using two protocols and crystal structures of TyrH (ID: 1TOH)^45^ and mushroom Tyr (PDB ID: 5M6B)^48^ deposited in PDB^56^. Docking to the active sites the FlexX docking algorithm^57^ of the LeadIT environment as implemented in the LeadIT 2.1.9 program (BioSolveIT GmbH, Augustin, Germany) was employed. In the case of all analysed compounds, the first 100 top-ranked docking poses were saved for each docking run. In the search for allosteric binding sites the EADock DSS algorithm^58^, as implemented in the SwissDock^59^ program. In the cluster analysis, the radius of 2 Å and the maximum cluster size of 8 entries were used. Graphical representation of the docking results was conducted using the Chimaera software^60^.

#### Sequence alignments

Sequence alignments were created using MUSCLE multiple alignment software^47^. The catalytic domain of three enzymes from biopterin-dependent aromatic amino acid hydroxylase family (EC 1.14.16.) with tyrosine 3-monooxygenase activity were used: two aromatic amino acid hydroxylase of *T. gondii* (GenBank: TgAaaH1 – ACB99413.1) and TyrH from rat (GenBank: 1TOH – AAA42257.1). The conserved VPWFPR motif was identified as the beginning of the catalytic domain of enzymes^12^^,^^61^.

#### Graphs and statistical analyses

Statistical analyses and graphs were performed using GraphPad Prism version 9.0.0 on macOS (GraphPad Software, San Diego, CA). Compounds with CC_30_ or IC_50_ values greater than the highest concentration tested, they were calculated based on extrapolation of the curves using the GraphPad Prism. Additionally, to establish a relationship between cytotoxicity and antiparasitic activity, the SR values were calculated as the ratio of 30% cytotoxic concentration (CC_30_) to 50% antiparasitic concentration (IC_50Tg_).

## Conclusion

Herein, we have described anti-proliferation effects of two series of 4-arylthiosemicarbazides with imidazole or cyclopentane substitution at N1 position on highly virulent *T. gondii* RH strain. Among these series, compounds with *meta*-iodo substitution **6a** and **6b** showed efficacy close to TRI while exhibiting enhanced selectivity. Thus, compounds **6a** and **6b** could be used as lead structures in further structural optimisation and structure-activity relationship studies. Further experiments with compounds of both series demonstrated their inhibitory effects on Tyr activity, with good correlation being observed (Pearson correlation coefficient *r* = 0.84; *p* = .0053) between percentage of Tyr inhibition and IC_50Tg_ of both sets of thiosemicarbazides. Next, the most potent Tyr inhibitors, compounds **2a**, **2b**, **6a**, and **6b**, were explored to determine their efficacy in inhibiting *T. gondii* growth in human foreskin fibroblast cells using confocal microscopy. All studied compounds significantly reduced the number of tachyzoites in the PVs compared to untreated cells, and inhibited tachyzoites growth by impeding their division. In addition, the compounds effectively inhibited tachyzoites penetration into host cells. Collectively, the obtained results support the conclusion that the examined compounds with thiosemicarbazide scaffold were able of disrupting L-tyrosine metabolism in *Toxoplasma* tachyzoites by deregulation of TyrH – the crucial enzyme in the conversion of L-tyrosine to L-DOPA. The mechanism of the inhibition of this step included the binding to an allosteric site, which was in agreement with the mechanism recently reported for tryptophan hydroxylase.

## Supplementary Material

Supplemental Material
