# Supplementary material for: 4-Arylthiosemicarbazide derivatives as a new class of tyrosinase inhibitors and anti-Toxoplasma gondii agents
Source: J Enzyme Inhib Med Chem. 2021 Jun 2;36(1):1145–64. doi: 10.1080/14756366.2021.1931164 (PMC8174488; doi:10.1080/14756366.2021.1931164)
Supplement: Supplemental Material [file IENZ_A_1931164_SM1267.pdf]

Supplementary Material

4-Arylthiosemicarbazide derivatives as a new class of tyrosinase inhibitors and anti-*Toxoplasma gondii* agents

Adrian Bekier[a], Lidia Węglińska[b], Agata Paneth[b]*, Piotr Paneth[c,d] and Katarzyna Dzitko[a]*

[a] *Department of Molecular Microbiology, Faculty of Biology and Environmental Protection, University of Lodz, Lodz, Poland;* [b] *Department of Organic Chemistry, Faculty of Pharmacy, Medical University of Lublin, Lublin, Poland;* [c] *Institute of Applied Radiation Chemistry, Lodz University of Technology, Lodz, Poland;* [d] *Institute Center for Research on Innovative Biobased Materials (ICRI-BioM) – International Research Agenda, Lodz University of Technology, Lodz, Poland*

Table of contents:

The cytotoxic concentration (CC_{30}) of thiosemicarbazide derivatives against Hs27 cell line.

^{1}H and ^{13}C spectra of representative model compounds.

Table 1. The cytotoxic concentration (CC_{30}) of **1a-9a** and **1b-9b** against Hs27 cell line.

Compound	CC_{30}[a]	Compound	CC_{30}[a]
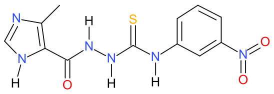 **1a**	479.22	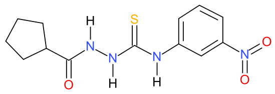 **1b**	603.98
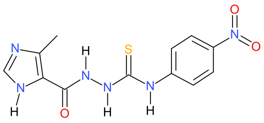 **2a**	697.67	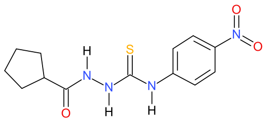 **2b**	405.09
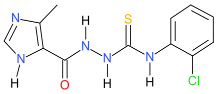 **3a**	502.21	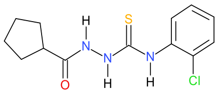 **3b**	512.31
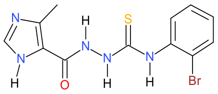 **4a**	728.41	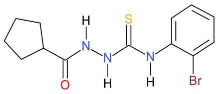 **4b**	>1000
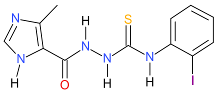 **5a**	278.72	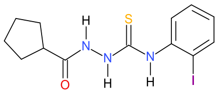 **5b**	369.69
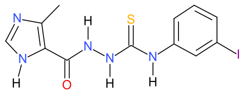 **6a**	209.45	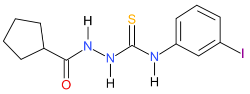 **6b**	135.87
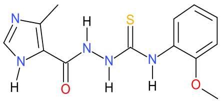 **7a**	498.12	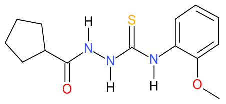 **7b**	>1000
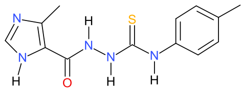 **8a**	421.89	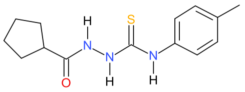 **8b**	445.10
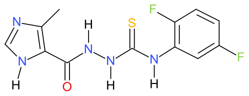 **9a**	612.48	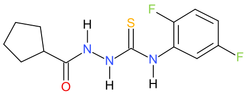 **9b**	>1000
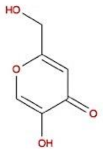 Kojic acid (**KA**)	>2500		

[a]CC_{30} [μM] represents the non-cytotoxic concentration of the compound required for 30% of cells proliferation inhibition *in vitro*. CC_{30} values were determined based on the plotted curves.

1b

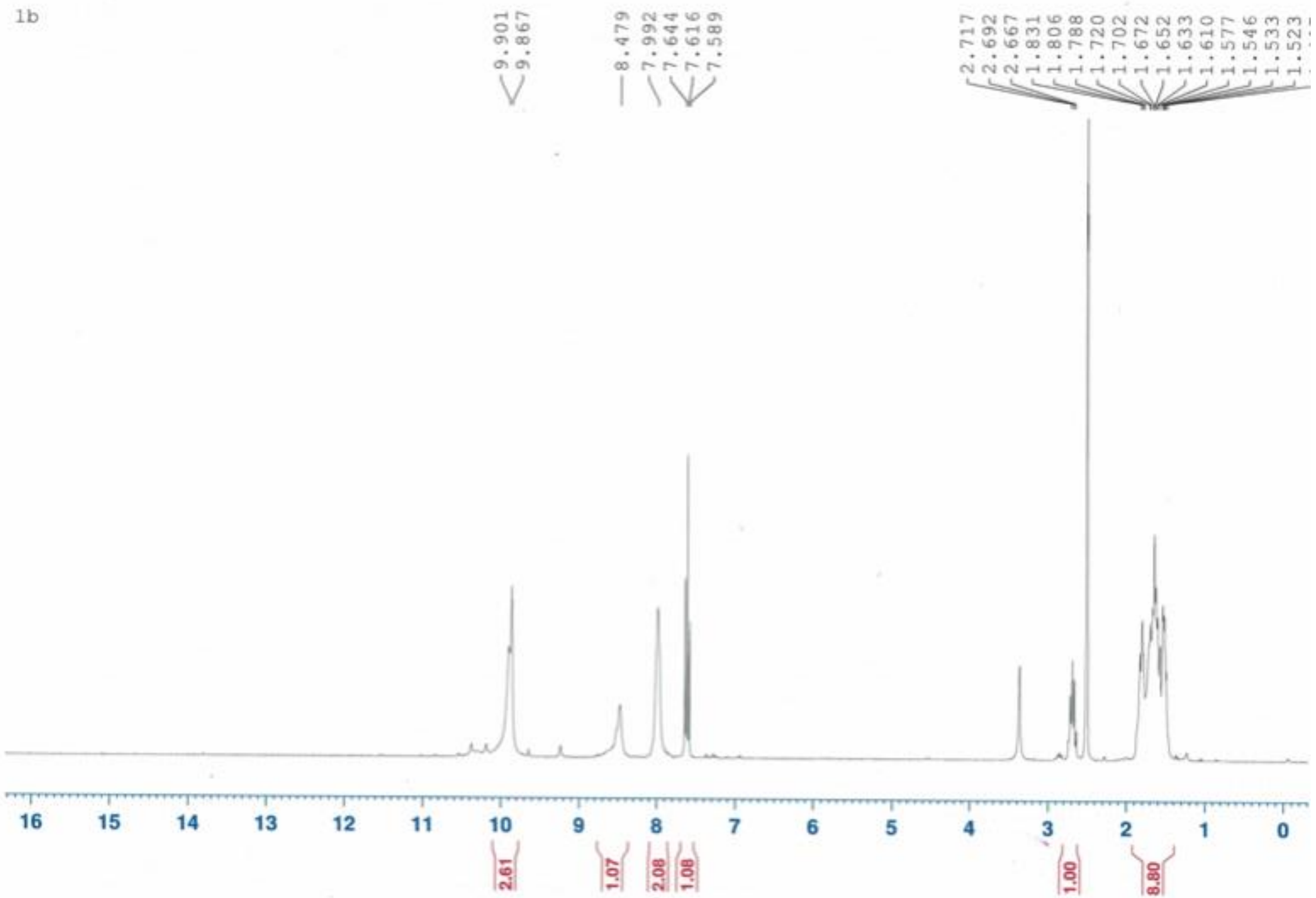
9.901
9.867
8.479
7.992
7.644
7.616
7.589
2.717
2.692
2.667
1.831
1.806
1.788
1.720
1.702
1.672
1.652
1.633
1.610
1.577
1.546
1.533
1.523
16
15
14
13
12
11
10
9
8
7
6
5
4
3
2
1
0
2.61
1.07
2.08
1.08
1.00
8.80

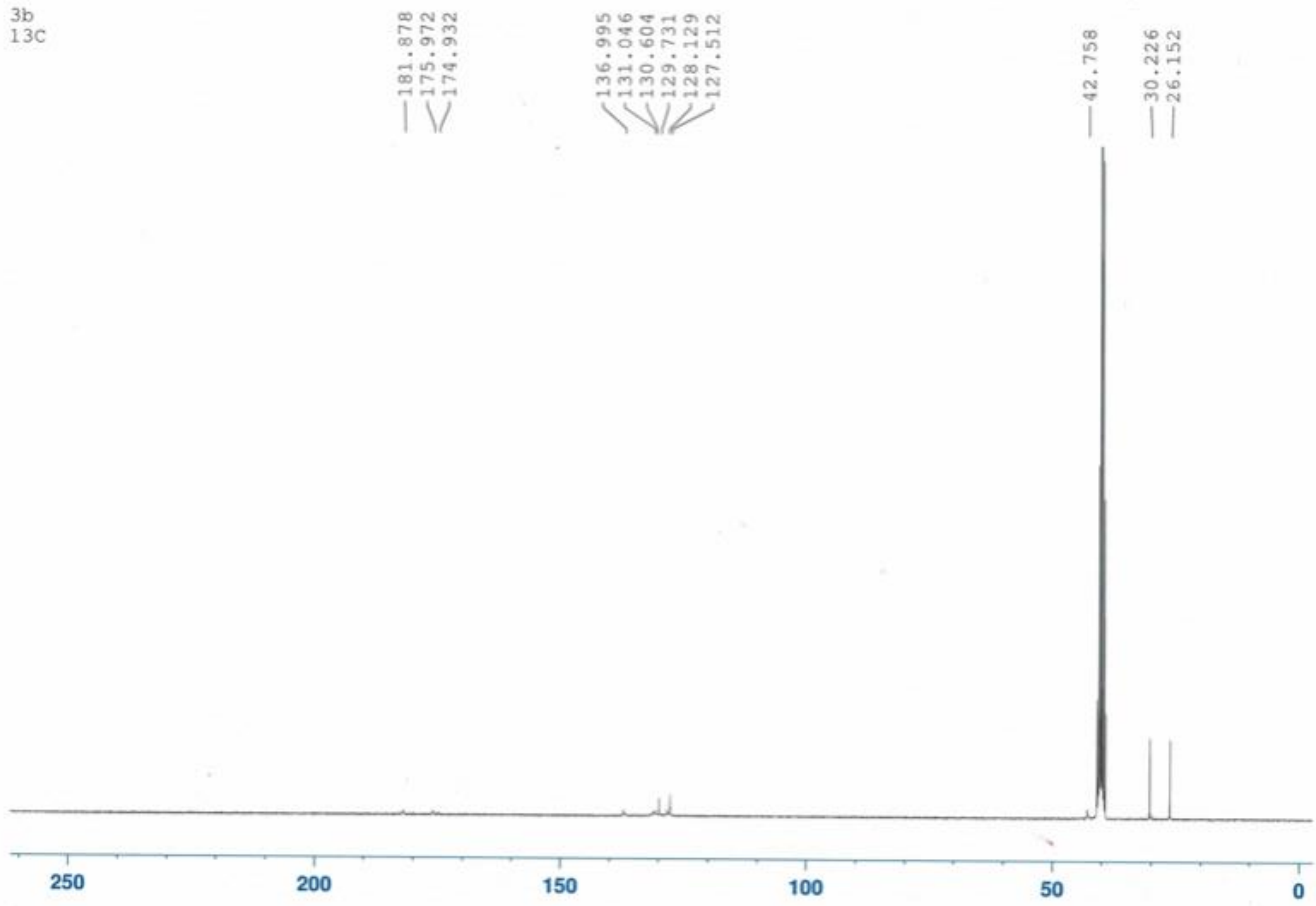
3b
13C
181.878
175.972
174.932
136.995
131.046
130.604
129.731
128.129
127.512
42.758
30.226
26.152
250
200
150
100
50
0

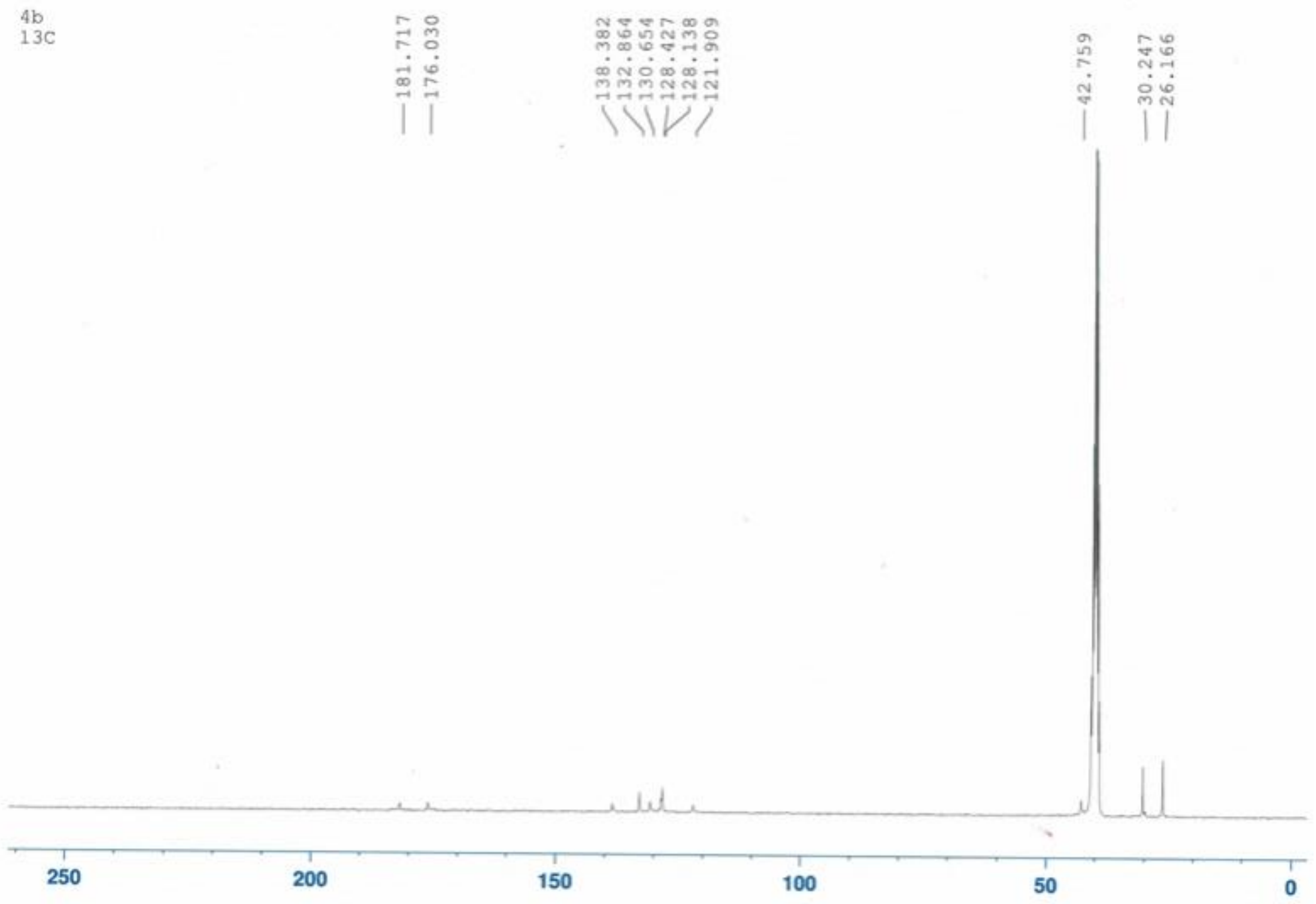
4b
13C
181.717
176.030
138.382
132.864
130.654
128.427
128.138
121.909
42.759
30.247
26.166
250
200
150
100
50
0

5b

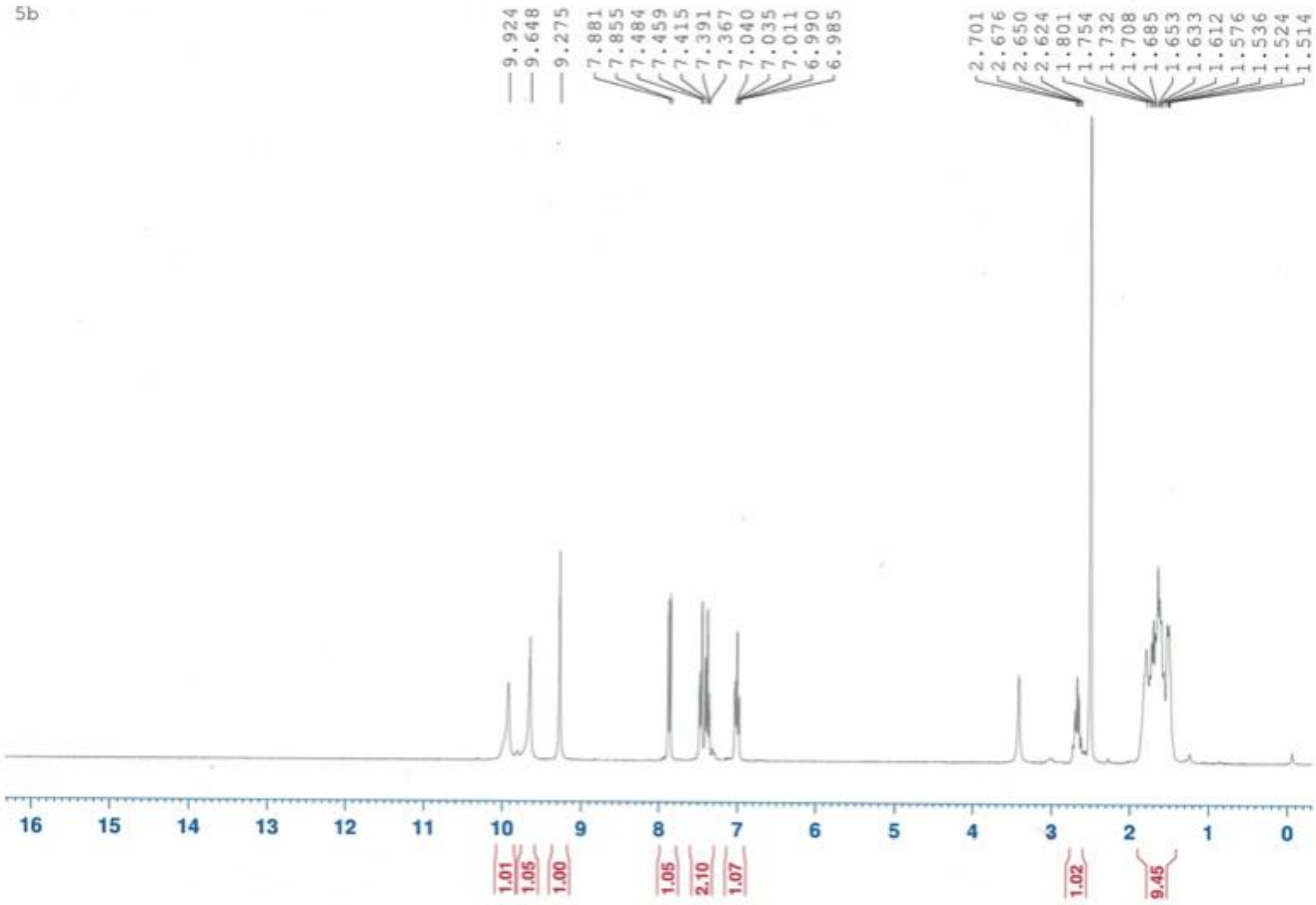

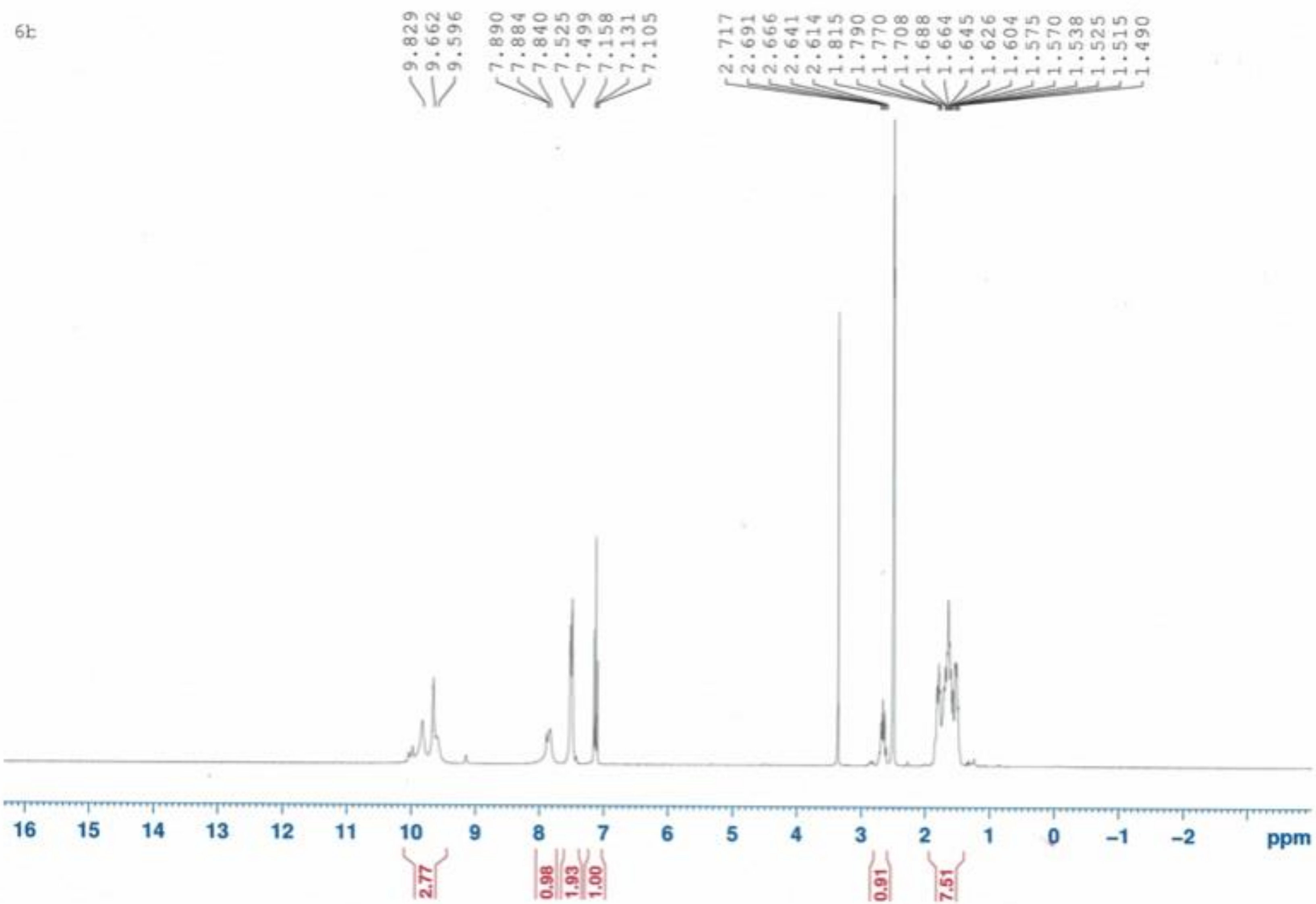
6t
9.829
9.662
9.596
7.890
7.884
7.840
7.525
7.499
7.158
7.131
7.105
2.717
2.691
2.666
2.641
2.614
1.815
1.790
1.770
1.708
1.688
1.664
1.645
1.626
1.604
1.575
1.570
1.538
1.525
1.515
1.490
16 15 14 13 12 11 10 9 8 7 6 5 4 3 2 1 0 −1 −2 ppm
2.77
0.98
1.93
1.00
0.91
7.51

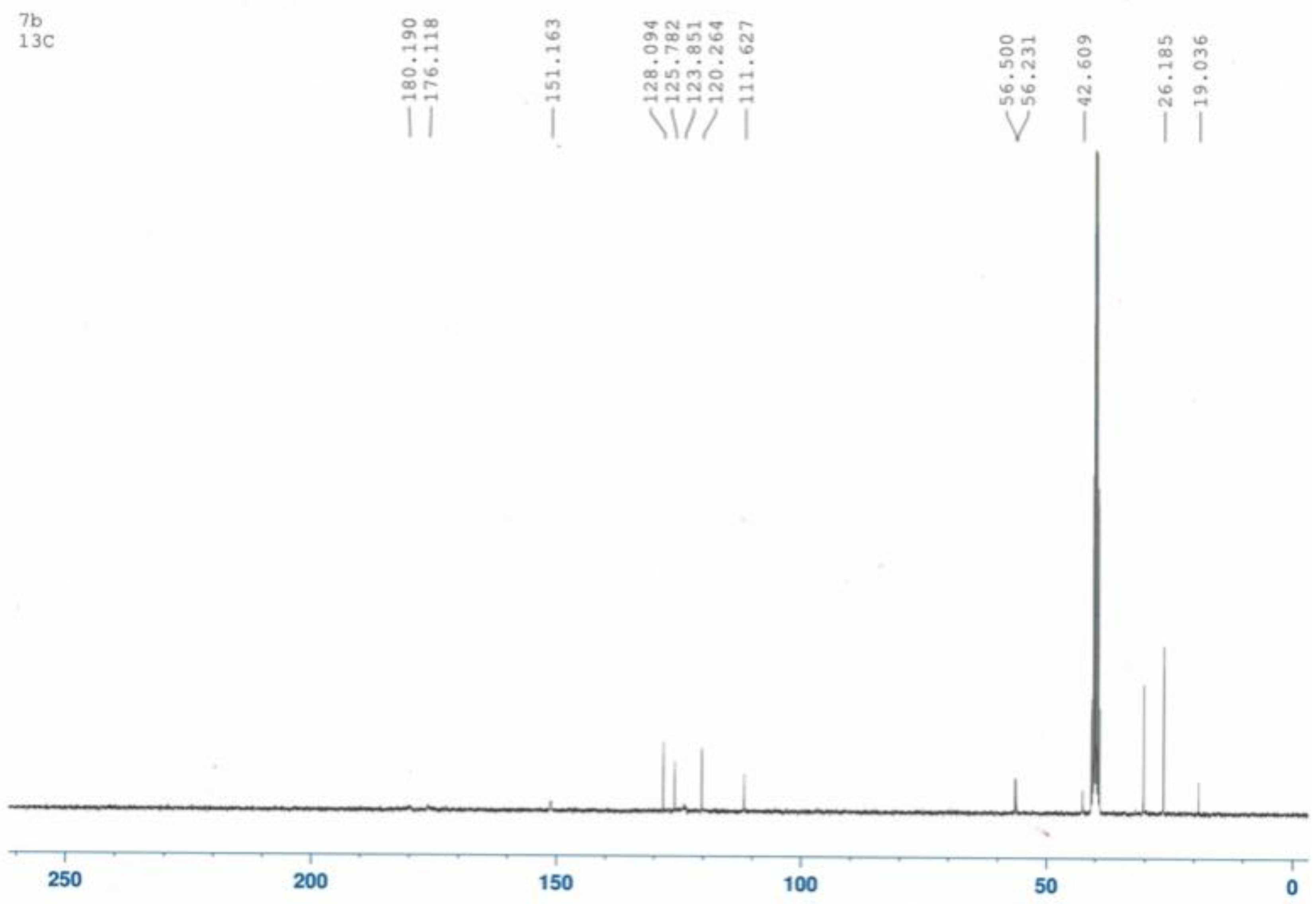
7b
13C
180.190
176.118
151.163
128.094
125.782
123.851
120.264
111.627
56.500
56.231
42.609
26.185
19.036
250
200
150
100
50
0

8b

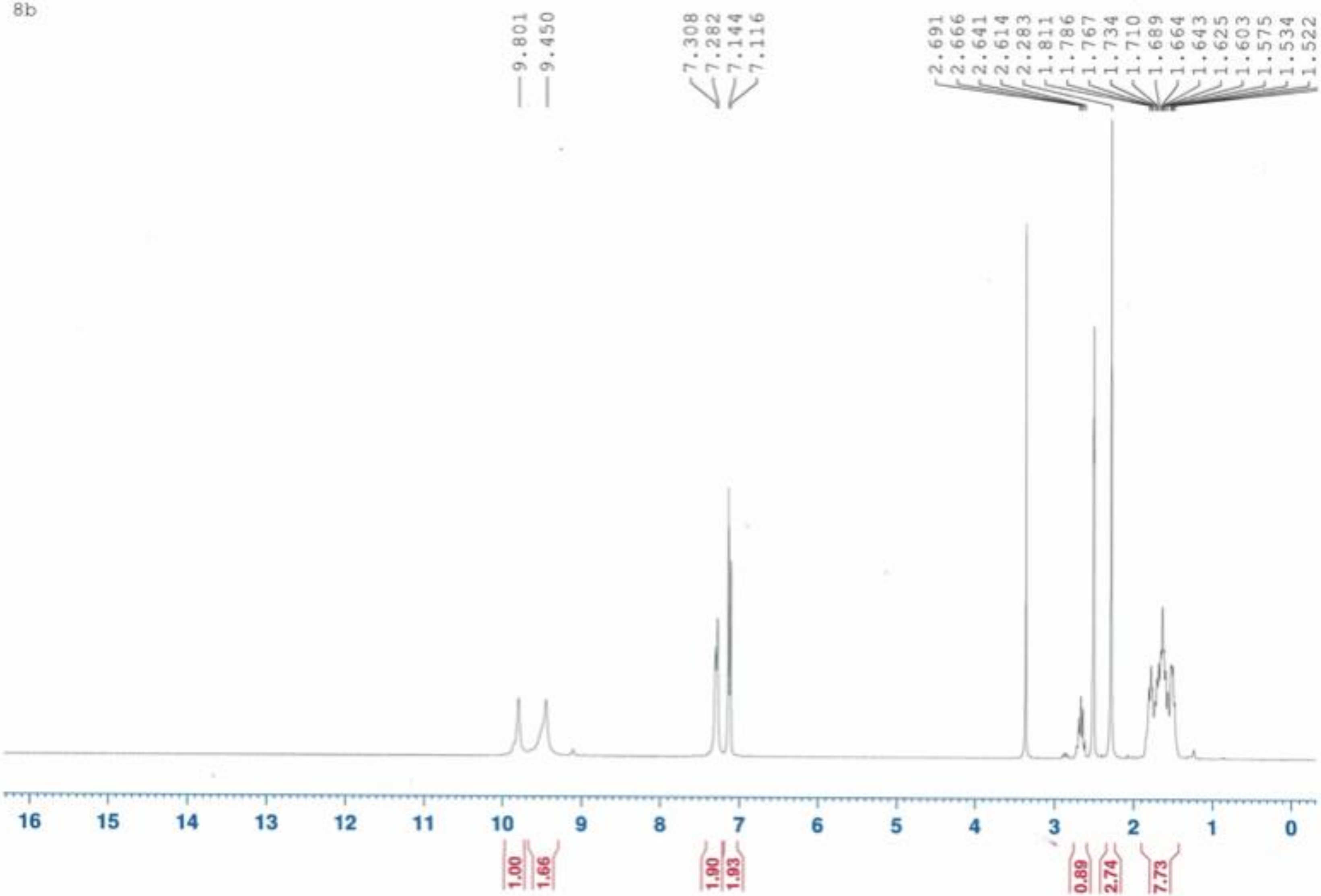
9.801
9.450
7.308
7.282
7.144
7.116
2.691
2.666
2.641
2.614
2.283
1.811
1.786
1.767
1.734
1.710
1.689
1.664
1.643
1.625
1.603
1.575
1.534
1.522
16 15 14 13 12 11 10 9 8 7 6 5 4 3 2 1 0
1.00
1.66
1.90
1.93
0.89
2.74
7.73
